# Distinct dynamical and thermodynamic pathways compound to amplify storylines of extreme atmospheric rivers in British Columbia

**DOI:** 10.1038/s41612-026-01476-8

**Published:** 2026-07-05

**Authors:** Tim Whittaker, Alejandro Di Luca

**Affiliations:** Centre Étude et simulation du climat á l’échelle régionale (ESCER), Département des Sciences de la Terre et de l’Atmosphère, Université du Québec à Montréal, Montréal, QC Canada

**Keywords:** Climate sciences, Hydrology

## Abstract

Atmospheric rivers (ARs) over western North America drive extreme precipitation and flood hazard, yet their intensity upper bounds remain poorly constrained by the short observational record. We combine a differentiable global climate model (~1.4°) with high-resolution dynamical downscaling (~0.11°) to construct physically plausible storylines of five unprecedented AR events in British Columbia. By optimizing minimal perturbations to historical initial conditions, we generate events that maximize integrated vapor transport (IVT) and exceed the observational record under present-day conditions. Pseudo-global warming perturbations under SSP5–8.5 provide a second pathway through end-of-century warming. Both approaches amplify AR intensity through distinct mechanisms: the optimization primarily modifies the wind field, while the pseudo-global warming signal primarily increases atmospheric moisture. These contributions act on largely independent components, and when applied simultaneously, the combined effect is nearly additive, with differences generally below 15%. Non-linear effects, though small, are always positive, and within these storylines the most extreme physically plausible ARs arise from compounding both drivers. The pathways differ in precipitation efficiency: the dynamical amplification largely preserves the conversion of moisture transport to precipitation, whereas the thermodynamic amplification reduces it by up to 29%. Bounding the upper tail of AR hazard may therefore require accounting for both dynamical variability and thermodynamic change.

## Introduction

Atmospheric rivers (ARs) are long, narrow, and transient filaments of intense horizontal water vapor transport, responsible for the majority of poleward moisture transport in the midlatitudes^1,2^. ARs are also the dominant drivers of extreme precipitation and flooding along the west coasts of midlatitude continents. Their societal importance is twofold: they are critical for water resources, but they are also responsible for major flooding events^3^.

This dual role is especially pronounced in western North America. Along the coast of so-called British Columbia and southeastern Alaska, ARs contribute up to 33% of the total annual precipitation and account for over 90% of annual extreme precipitation events in the coastal mountains^4^ and are also linked with future increases in flood risk in some of the major river basins, such as the Fraser basin^5^. For instance, the November 2021 British Columbia flooding, which became the costliest natural disaster in the province’s history, was a compound event driven by a series of intense ARs, along with already saturated soils^6,7^. An attribution analysis of the 2021 event revealed that the two-day precipitation was approximately a 1-in-50 to 1-in-100-year event due in-part to human-induced climate change^7^. Single event attribution studies found that human-induced climate change has made such an event’s precipitation 45–60% more likely^7^ compared to pre-industrial conditions, consistent with findings from other events that climate change is enhancing AR precipitation^8^.

The standard metric for AR intensity, the vertically-integrated water vapor transport (denoted as *IVT*), couples the two primary physical components that define the storm: the thermodynamic component (*q*, or atmospheric water vapor content) and the dynamic component (**V**_*h*_, the horizontal wind field). The formation of most high-impact midlatitude ARs are closely linked to the life cycle of extratropical cyclones; ARs are typically formed by the cold front of a cyclone sweeping up water vapor within the warm sector and concentrating it at the base of the warm conveyor belt^9^. The ultimate impact is then determined at landfall, where the interaction of the wind field with the topography forces orographic lift and precipitation^10^. The specific orientation, or incident angle, of the AR relative to the mountain barrier is a critical factor in maximizing this uplift and subsequent precipitation^11,12^. In British Columbia, for instance, the direction of an approaching AR has been shown to determine the IVT threshold required to produce extreme flooding, with westerly-approaching ARs generating extreme events at substantially lower IVT magnitudes than those arriving from more southerly directions^13^.

Climate projections suggest that the primary source of more intense ARs in the future will be thermodynamic, as a warmer atmosphere holds more moisture (following the Clausius-Clapeyron relationship), leading to increases in IVT^14–16^. This thermodynamic contribution is relatively well-constrained by regional temperature changes, and its dominant role has been highlighted in studies that separate the effects from humidity in IVT^16^. However, the potential for amplification from the dynamic component of ARs is less certain. Indeed, recent studies analysing the historical record have yielded mixed results regarding trends in AR intensification^17,18^. The track, frequency, and intensity of ARs are heavily modulated by large-scale circulation anomalies like the Pacific-North American (PNA) pattern or Rossby wave breaking^3,19,20^. It remains unknown whether the most extreme, plausible ARs are simple thermodynamic amplifications of known dynamic events (e.g., wetter storms) or if they are driven by qualitatively different dynamic structures—rare configurations of the wind field that are physically possible but which we have not yet observed^21,22^.

This specific question has remained difficult to answer in part due to a fundamental sampling limitation. The observational record is too short to capture the full range of what is physically possible, leaving society vulnerable to potentially unknown rare and high-impact events. Conventional climate model simulation strategies, for example the single model initial-condition large ensembles, are computationally costly and are produced using relatively low horizontal resolution models, limiting the value of the simulations and providing insufficient, unguided sampling of the high-dimensional phase space required to observe these rare and specific events^23,24^. This is particularly an issue for events such as ARs where a high-resolution model is necessary to capture the physics of the precipitation. In these cases, large ensembles are simply intractable. This challenge has led to the development of event-based storylines, which focus on physical plausibility rather than probability, as a more robust way to assess risk in the face of uncertainty^25,26^.

The pseudo-global warming (PGW) method^27^, in which a climate change signal derived from global climate model (GCM) projections is superimposed on the lateral boundary conditions of historical events, has emerged as one of the most widely used tools for constructing such storylines for future extreme events. Mahoney et al.^28^ used the PGW method to examine an inland-penetrating AR that caused flooding across the U.S. Pacific Northwest, finding increased total precipitation in all future simulations, with ensemble-mean increases exceeding 150 mm across so-called western Oregon and Washington under RCP8.5 warming. Sandvik et al.^29^ applied idealized PGW perturbations to extreme orographic precipitation events driven by strong onshore moisture fluxes and found a mean rainfall increase of ~16% K^−1^ that was partly offset by a ~23% K^−1^ reduction in snowfall, attributing the limited net precipitation response in part to a shift from more efficient ice-phase precipitation growth to less efficient warm-rain processes. While effective at quantifying how known events intensify under warming, PGW does not address the complementary question of whether the same events could reach higher intensities under present-day conditions through different dynamical configurations.

To overcome the sampling barrier and generate these extreme event storylines, new computational methods have been developed. One family of approaches, known as ensemble boosting, guides simulations toward a high-impact outcome by iteratively re-sampling and perturbing ensemble members^24,30,31^. Other methods apply techniques from statistical physics, such as large deviation theory or adaptive multilevel splitting algorithms, to efficiently sample the tails of the distribution and compute probabilities for extreme heat and rainfall^32–35^.

A more recent approach exploits the differentiability of modern data-driven and hybrid forecasting models, where hybrid refers to architectures that combine a traditional dynamical core with neural network parametrizations of subgrid processes. Because these models are end-to-end differentiable, backpropagation can be used to compute gradients of model outputs with respect to initial conditions^36^. This enables optimization algorithms to find minimal and physically plausible initial conditions perturbations required leading to a specific high-impact event^37,38^. Recent benchmarking has demonstrated that data-driven models can accurately represent AR dynamics^39^, with the hybrid model NeuralGCM (NGCM) showing particular skill in simulating AR intensity and structure^40^.

Here, we apply this optimization framework to construct storylines of more extreme ARs in western Canada in order to characterize the physical pathways through which such amplification can occur. We combine the NeuralGCM (NGCM) differentiable hybrid global climate model with high-resolution regional modelling to construct and physically decompose unprecedented AR storylines. Five historical AR events that made landfall along the coast of British Columbia, selected from the EDARA catalogue^41^ as the most intense by mean integrated vapor transport (IVT) over a coastal target region, are used to initialize 70-h forecasts. For each event we run a control forecast (CTRL) and an optimized forecast (OPT) in which gradient-based optimization identifies the smallest perturbation to the initial atmospheric state that maximizes IVT over the target region during a 24–54 h analysis window. Both forecasts are then dynamically downscaled with the CRCM6-GEM5 regional climate model to resolve the orographic interactions at landfall. This multi-model approach allows us to efficiently optimize the large-scale circulation using the differentiable global model while resolving precipitation at high resolution. An additional pair of configurations, CTRL_PGW_ and OPT_PGW_, repeat the downscaling with a pseudo-global warming (PGW) perturbation derived from a CMIP6 SSP5–8.5 ensemble added to the lateral and surface boundary conditions, providing a thermodynamic end-of-century counterpart to each present-climate simulation. Comparing these four configurations isolates dynamical amplification under the present climate, thermodynamic amplification under future warming, and their combination, and allows us to test whether the two pathways act independently or compound.

## Results

### Control simulations and event characteristics

**Fig. 1:**
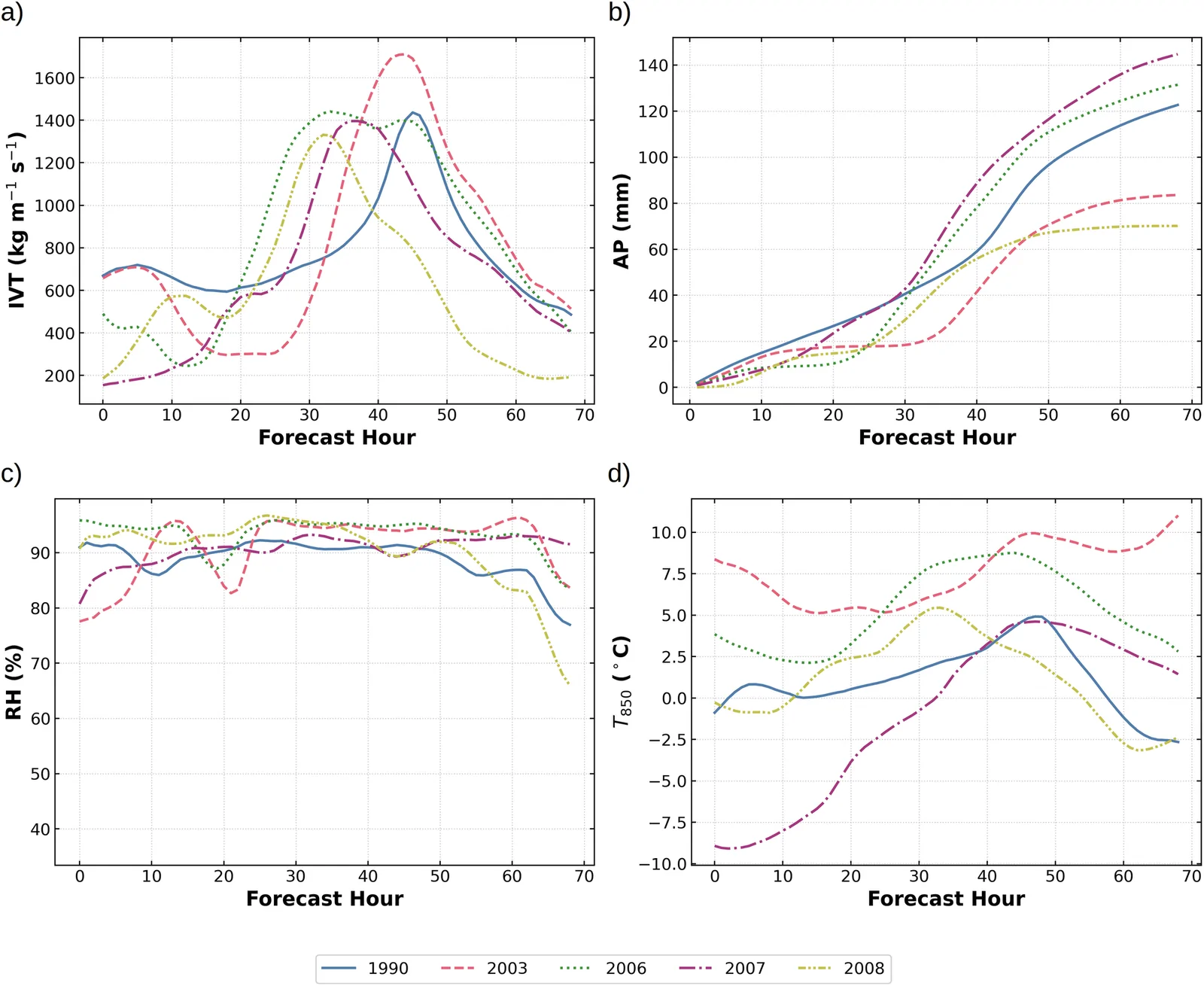
Control simulation (CTRL) regional-mean time series for the five selected AR events. Event years: 1990, solid blue; 2003, dashed red; 2006, dotted green; 2007, dash-dot purple; 2008, dash-dot-dot yellow. **a** Integrated vapor transport (IVT; kg m^−1^ s^−1^), **b** accumulated precipitation (AP; mm), **c** column-mean relative humidity (%), and **d** 850 hPa temperature (°C).

Five significant AR events that made landfall along the coast of British Columbia were selected from the EDARA catalogue (see Methods) and downscaled using CRCM6-GEM5 driven by NGCM boundary conditions of the simulated events, producing 70-h control simulations. Figure 1 presents the regional-mean temporal evolution of these simulations.

All five events reach extreme IVT magnitudes exceeding 1300 kg m^−1^ s^−1^ (Fig. 1a), with peak values occurring between hours 30 and 45 after the beginning of the simulation, and event durations generally between 10 and 20 h. The 2003 event is the most intense AR in terms of IVT, peaking near 1700 kg m^−1^ s^−1^ around hour 45, while the 1990 event sustains a broad maximum near 1400 kg m^−1^ s^−1^ between hours 40 and 55. The 2006 event is distinctive in exhibiting a double-peaked structure with two IVT maxima near hours 30 and 50, suggestive of two successive moisture surges. The atmospheric column is near saturation throughout the AR lifecycle: column-mean relative humidity (Fig. 1c) remains above 90% during peak IVT for all events, with several sustaining values close to 95%.

The five selected events all occur between October and December, reflecting the climatology of the region, where the most intense ARs along the British Columbia coast are concentrated in October to February^4^. Ranking by mean IVT therefore yields a cool-season set by construction. Near-surface temperatures (Fig. 1d) are generally between 0 and 8 °C during peak IVT, with the 2003 event the warmest (rising to ~15 °C) and the 2007 event the coldest (beginning near −6 °C). All events show a temperature increase coincident with peak IVT, consistent with warm-air within the AR corridor. At these cool-season temperatures, the Clausius–Clapeyron relationship constrains the saturation vapor pressure well below warm-season values; combined with the near-saturated conditions, the cool-season atmosphere is nearly as moist as it can be. Accumulated precipitation (Fig. 1b) ranges from ~70 mm (2008 event) to 145 mm (2007 event), with the largest accumulation rates coinciding with peak IVT between hours 25 and 55.

**Fig. 2:**
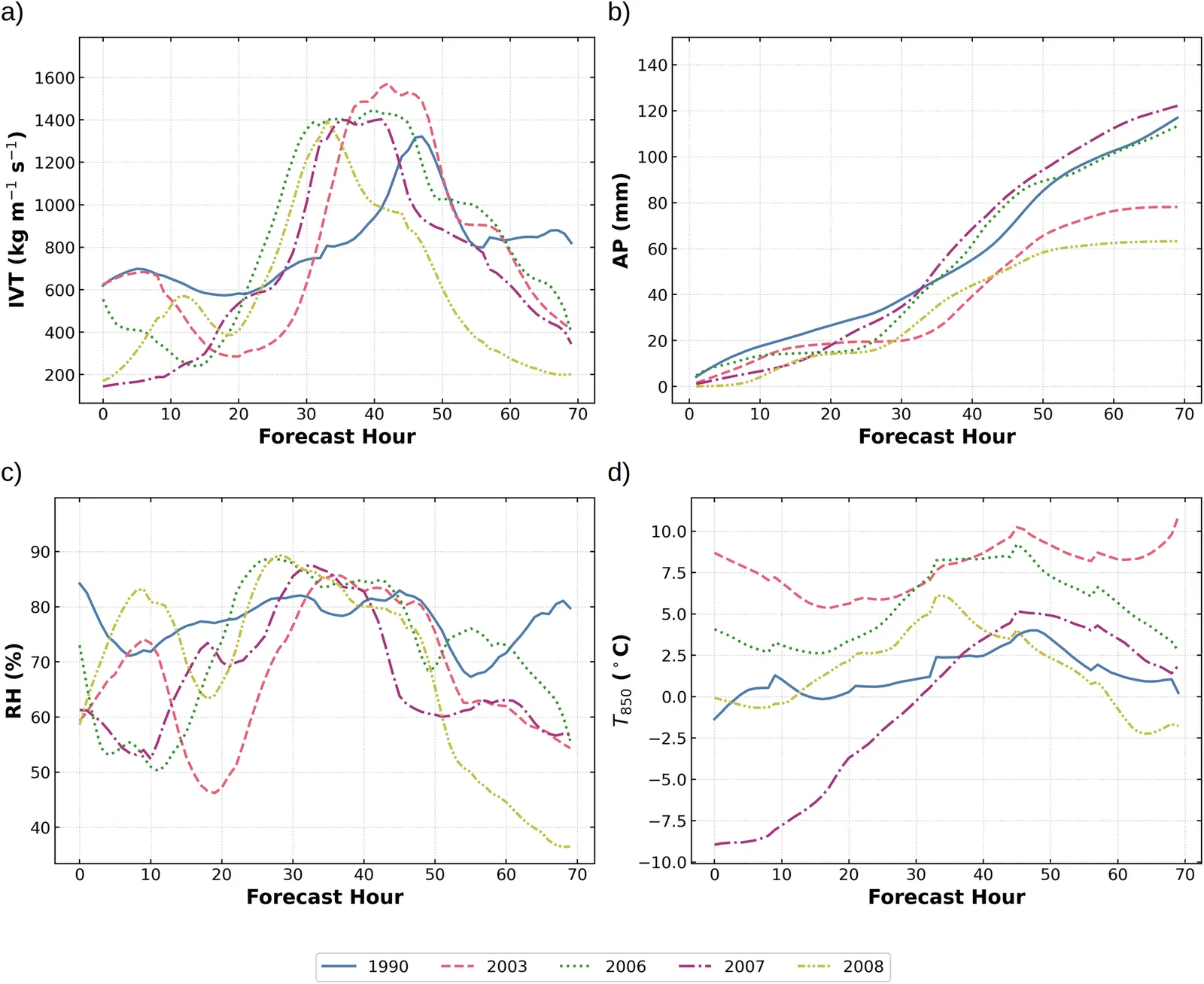
ERA5 regional-mean time series for the five selected AR events. Event years: 1990, solid blue; 2003, dashed red; 2006, dotted green; 2007, dash-dot purple; 2008, dash-dot-dot yellow. **a** IVT (kg m^−1^ s^−1^), **b** accumulated precipitation (AP; mm), **c** column-mean relative humidity (%), and **d** 850 hPa temperature (°C).

The same regional-mean time series computed from ERA5 reanalysis for the five events are shown in Fig. 2. CTRL simulations capture the observed events well, with the timing and magnitude of peak IVT and the temporal evolution of 850 hPa temperature consistent with ERA5. A wet bias is present in column-mean relative humidity and accumulated precipitation; part of the relative humidity offset is definitional, as ERA5 uses a mixed-phase saturation reference while the CRCM6-GEM5 relative humidity field does not. Mean bias (MB) and root-mean-square error (RMSE) of CTRL relative to ERA5, pooled over all five events: (a) MB = −0.1, RMSE = 87.9 kg m^−1^ s^−1^; (b) MB = + 3.8, RMSE = 8.6 mm; (c) MB = + 20.3%, RMSE = 23.2%; (d) MB = −0.3, RMSE = 0.8 °C. The corresponding CTRL − ERA5 error time series are shown in Supplementary Fig. 8. The agreement on IVT and temperature is sufficient to establish the CTRL simulations as a meaningful baseline for the amplification experiments that follow.

### IVT amplification through the NGCM optimized solution

**Fig. 3:**
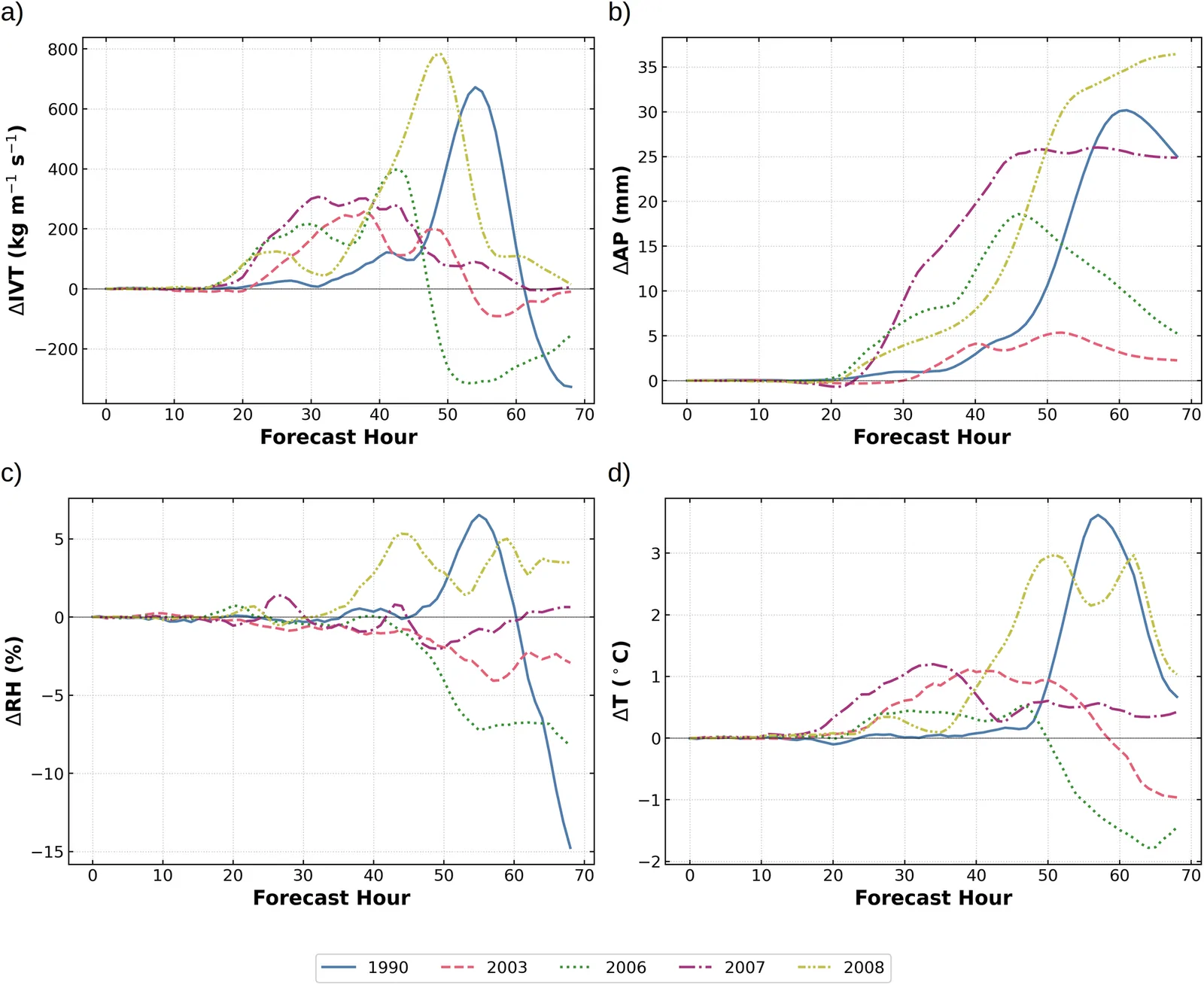
Response to the optimized perturbation for the dynamical perturbation experiment (OPT–CTRL). Event years: 1990, solid blue; 2003, dashed red; 2006, dotted green; 2007, dash-dot purple; 2008, dash-dot-dot yellow. **a** *Δ*IVT (kg m^−1^ s^−1^), **b** *Δ*AP (mm), **c** *Δ*RH (%), and **d** *ΔT* (°C).

The optimization amplifies IVT across all five events (Fig. 3a), with negligible effects during the first 10–20 forecast hours as the modified large-scale circulation develops. Peak *Δ*IVT (*IVT*_*OPT*_ − *IVT*_*CTRL*_) values range from ~300 kg m^−1^ s^−1^ (2007) to nearly 780 kg m^−1^ s^−1^ (2008), occurring between hours 35 and 55. This amplified moisture transport translates into increased precipitation for all events (Fig. 3b), with accumulated gains of 2–37 mm by the end of the simulation. The precipitation response lags the IVT amplification by several hours, as expected given the time required for orographic lifting and condensation to respond to the enhanced moisture flux. The optimized simulations are generally warmer by 1–3 °C (Fig. 3d), consistent with warmer air within the strengthened AR corridor, while relative humidity changes (Fig. 3c) are near zero during the first 30 h before diverging across events.

**Fig. 4:**
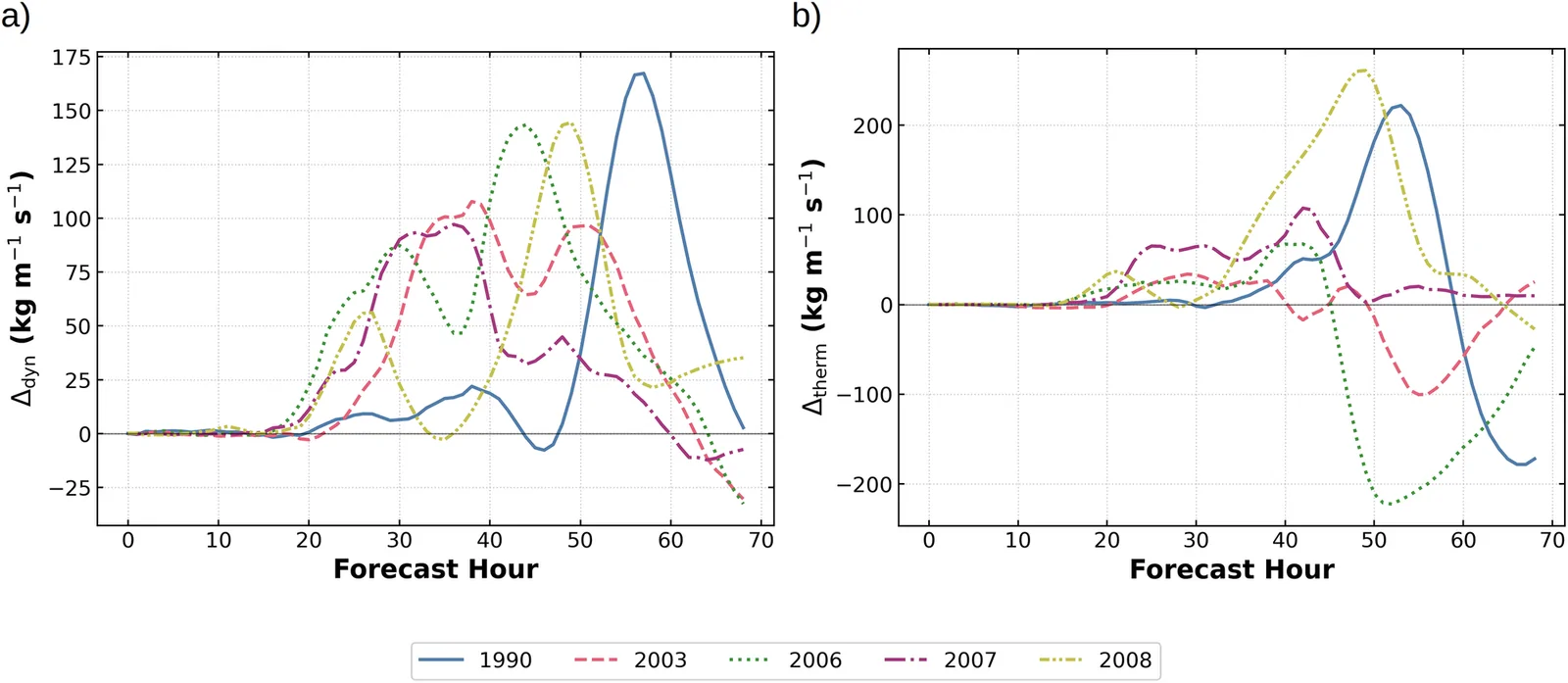
IVT decomposition for the dynamical perturbation experiment (OPT–CTRL), following Eqs. (7) and (8). Event years: 1990, solid blue; 2003, dashed red; 2006, dotted green; 2007, dash-dot purple; 2008, dash-dot-dot yellow. **a** Dynamic component *Δ*_dyn_ (kg m^−1^ s^−1^) and **b** thermodynamic component *Δ*_therm_ (kg m^−1^ s^−1^).

The decomposition of the IVT changes into dynamic (*Δ*_dyn_) and thermodynamic (*Δ*_therm_) components (see Methods) (Fig. 4) reveals that the amplification during the core phase of the AR event (defined as the period of peak IVT in the control simulations) is primarily driven by the dynamic component (Fig. 4a), which peaks at 190–325 kg m^−1^ s^−1^ between hours 30 and 45. The thermodynamic component (Fig. 4b) is usually secondary during this core period but, for two of the events, grows in magnitude during the later forecast hours, reaching 500 kg m^−1^ s^−1^ for the 2008 event and dropping below −400 kg m^−1^ s^−1^ for the 2006 event after hour 55. This delayed thermodynamic response is consistent with the warmer conditions in the optimized AR corridor (Fig. 3d): with the atmospheric column remaining near saturation, the higher temperatures imply a higher saturation vapor pressure and therefore greater moisture content, effectively extending the duration and intensity of the event beyond its historical envelope. The dynamic restructuring of the wind field is thus the primary driver of IVT amplification during the observed event, while the thermodynamic response acts as a secondary, lagged amplifier that prolongs the moisture transport.

### IVT amplification through the PGW perturbations

**Fig. 5:**
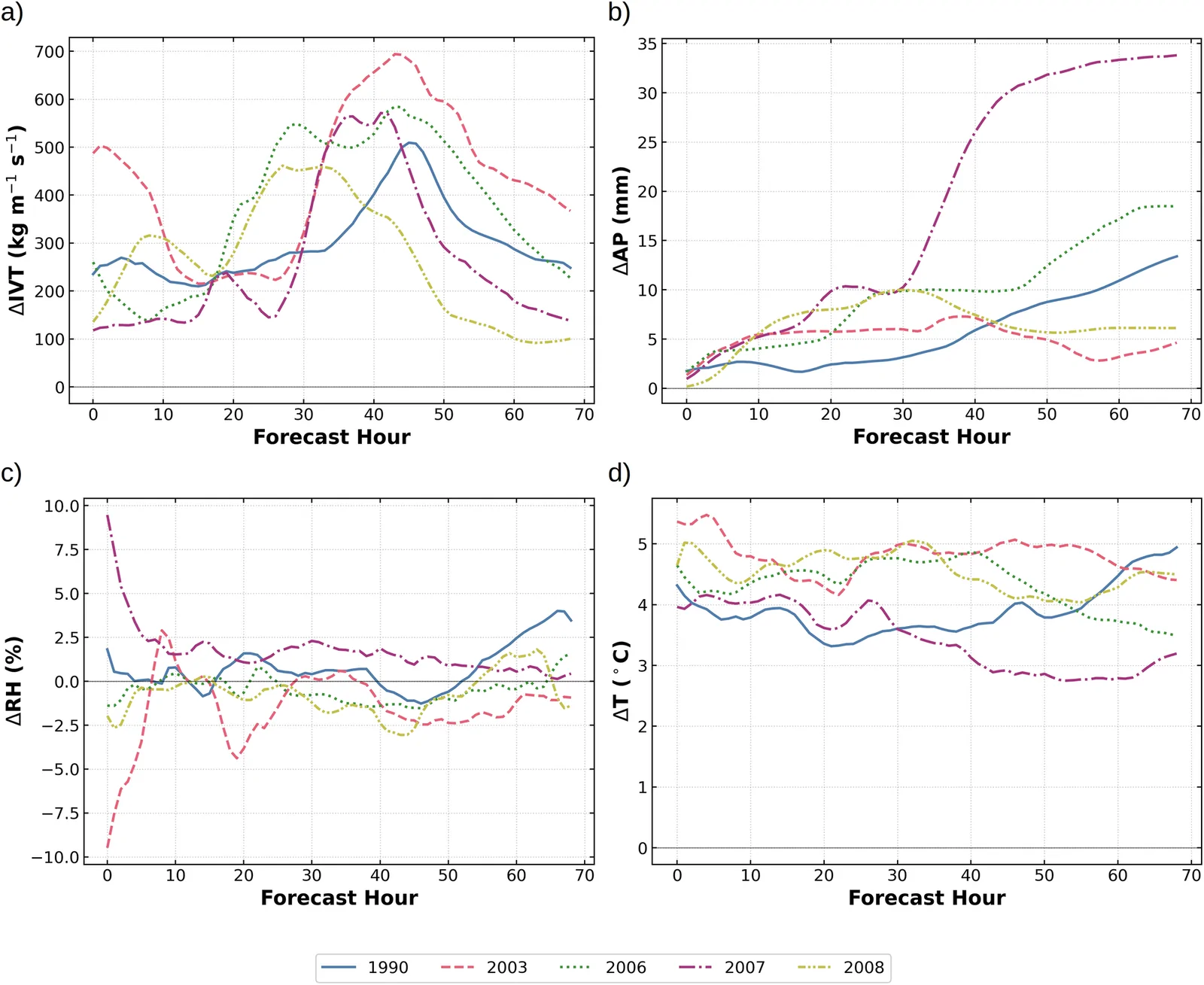
Regional-mean response to the PGW perturbation. As in Fig. 3, but for the PGW experiment (*CTRL*_*PGW*_–CTRL). Event years: 1990, solid blue; 2003, dashed red; 2006, dotted green; 2007, dash-dot purple; 2008, dash-dot-dot yellow. **a** *Δ*IVT (kg m^−1^ s^−1^), **b** *Δ*AP (mm), **c** *Δ*RH (%), and **d** *ΔT* (°C).

The PGW experiment produces on average larger *Δ*IVT (Fig. 5a), with all events showing positive anomalies peaking at 700 kg m^−1^ s^−1^ between hours 30 and 45. The precipitation response also shows a net positive increase (Fig. 5b) ranging from ~5 mm to ~35 mm. The *Δ*RH response (Fig. 5c) is small and mixed in sign. The temperature increase (*Δ*T) is consistent with the PGW superimposed climate change signal and shows little change during the event.

**Fig. 6:**
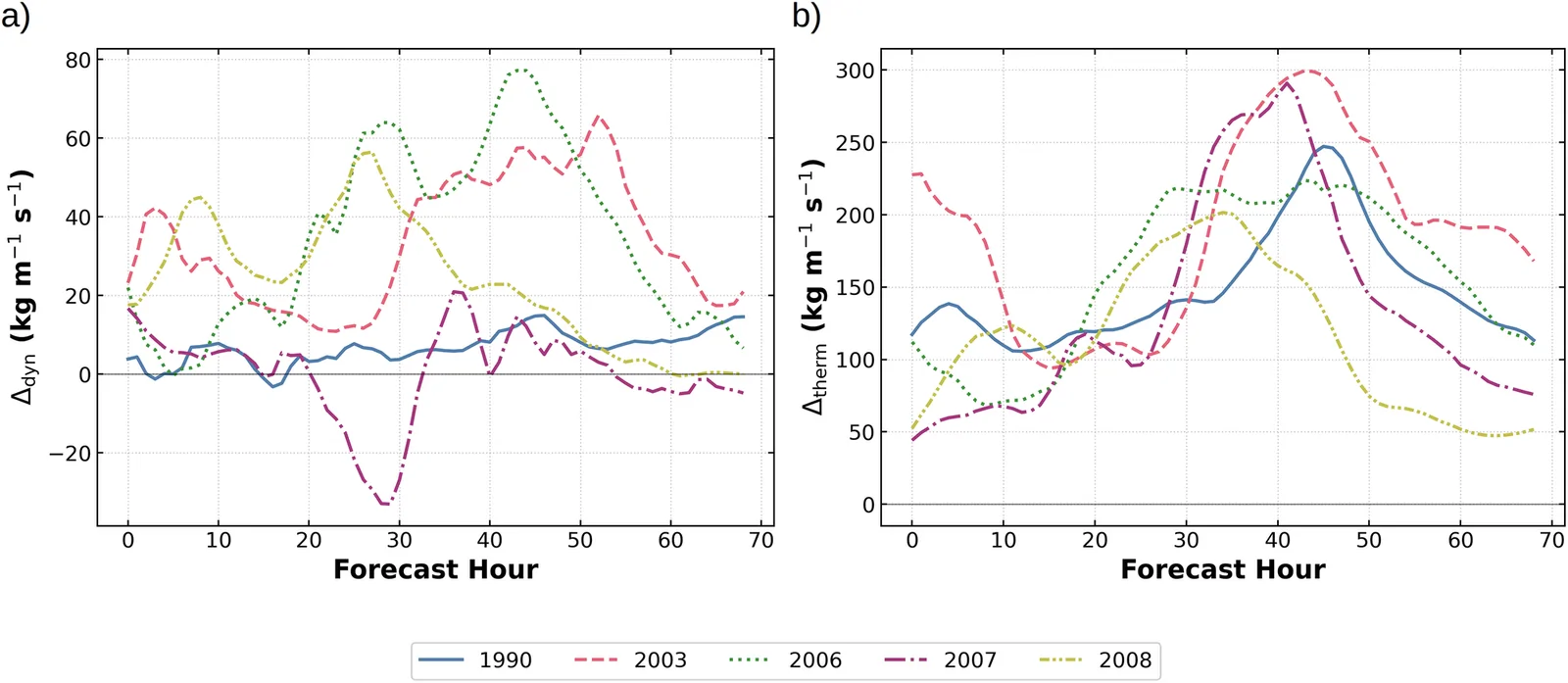
IVT decomposition for the PGW experiment. As in Fig. 4, but for the PGW experiment (*CTRL*_*PGW*_–CTRL). Event years: 1990, solid blue; 2003, dashed red; 2006, dotted green; 2007, dash-dot purple; 2008, dash-dot-dot yellow. **a** Dynamic component *Δ*_dyn_ (kg m^−1^ s^−1^) and **b** thermodynamic component *Δ*_therm_ (kg m^−1^ s^−1^).

The IVT decomposition (Fig. 6) shows a fundamentally different driving mechanism in the PGW compared to the optimized simulations. *Δ*_therm_ (Fig. 6b) is positive for all events, reaching peak values between 390 kg m^−1^ s^−1^ and 585 kg m^−1^ s^−1^. In contrast, while *Δ*_dyn_ (Fig. 6a) is also generally positive, it fluctuates within a much narrower range, between −60 and +150 kg m^−1^ s^−1^. The PGW amplification is thus primarily driven by the thermodynamic term, consistent with the Clausius–Clapeyron scaling of specific humidity under warming.

### IVT amplification through both optimization and PGW perturbations

**Fig. 7:**
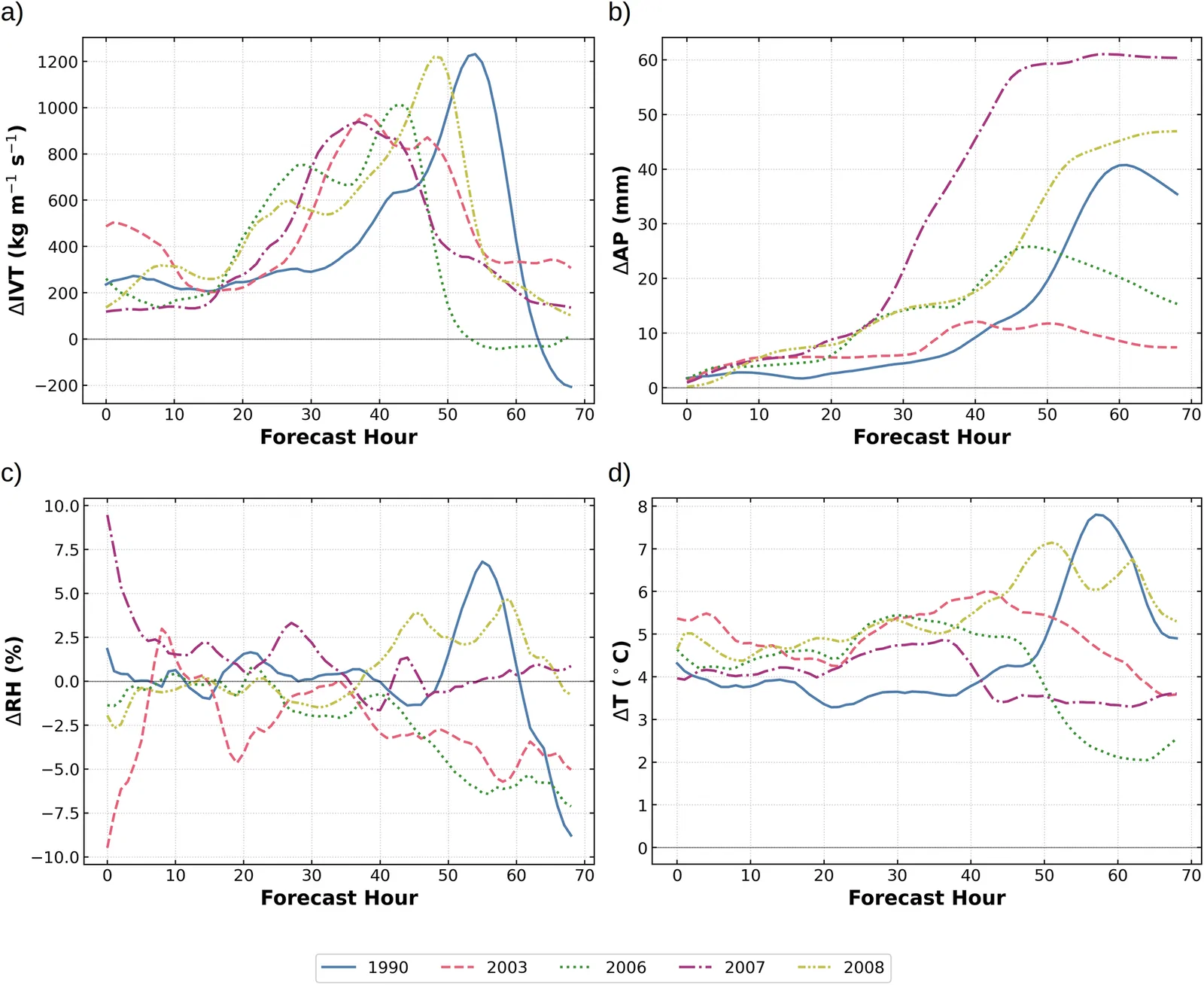
Regional-mean response to the combined perturbation. As in Fig. 3, but for the combined experiment (*OPT*_*PGW*_). Event years: 1990, solid blue; 2003, dashed red; 2006, dotted green; 2007, dash-dot purple; 2008, dash-dot-dot yellow. **a** *Δ*IVT (kg m^−1^ s^−1^), **b** *Δ*AP (mm), **c** *Δ*RH (%), and **d** *ΔT* (°C).

**Fig. 8:**
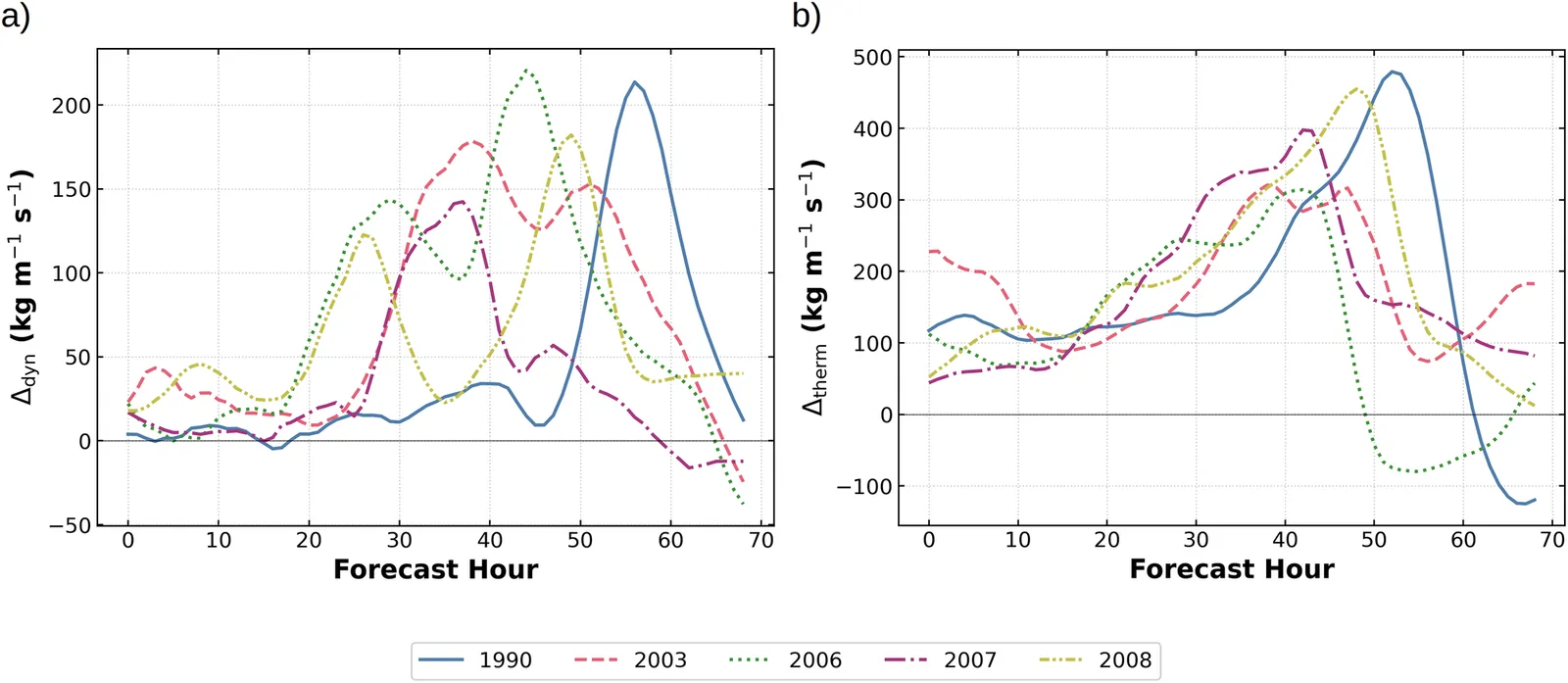
IVT decomposition for the combined experiment. As in Fig. 4, but for the combined experiment (*OPT*_*PGW*_). Event years: 1990, solid blue; 2003, dashed red; 2006, dotted green; 2007, dash-dot purple; 2008, dash-dot-dot yellow. **a** Dynamic component *Δ*_dyn_ (kg m^−1^ s^−1^) and **b** thermodynamic component *Δ*_therm_ (kg m^−1^ s^−1^).

When both perturbations are applied simultaneously (Fig. 7), *Δ*IVT reaches values between 930 kg m^−1^ s^−1^ and 1230 kg m^−1^ s^−1^ during hours 35 and 55 (Fig. 7a), exceeding either experiment alone. The precipitation response remains positive for all events (Fig. 7b), ranging from 8 mm to 60 mm. IVT decomposition terms show that both components contribute to the increase, with the thermodynamic component leading in intensity (Fig. 8): *Δ*_dyn_ peaks at 276–415 kg m^−1^ s^−1^, retaining the structure of the OPT-only case, while *Δ*_therm_ peaks at 610–931 kg m^−1^ s^−1^, exceeding the PGW-only values. The latter follows from the interaction between the two perturbations: in the combined experiment the stronger optimized winds act on the larger PGW moisture increment, so *Δ*_therm_, the moisture transported by the mean flow, exceeds the PGW signal alone.

**Fig. 9:**
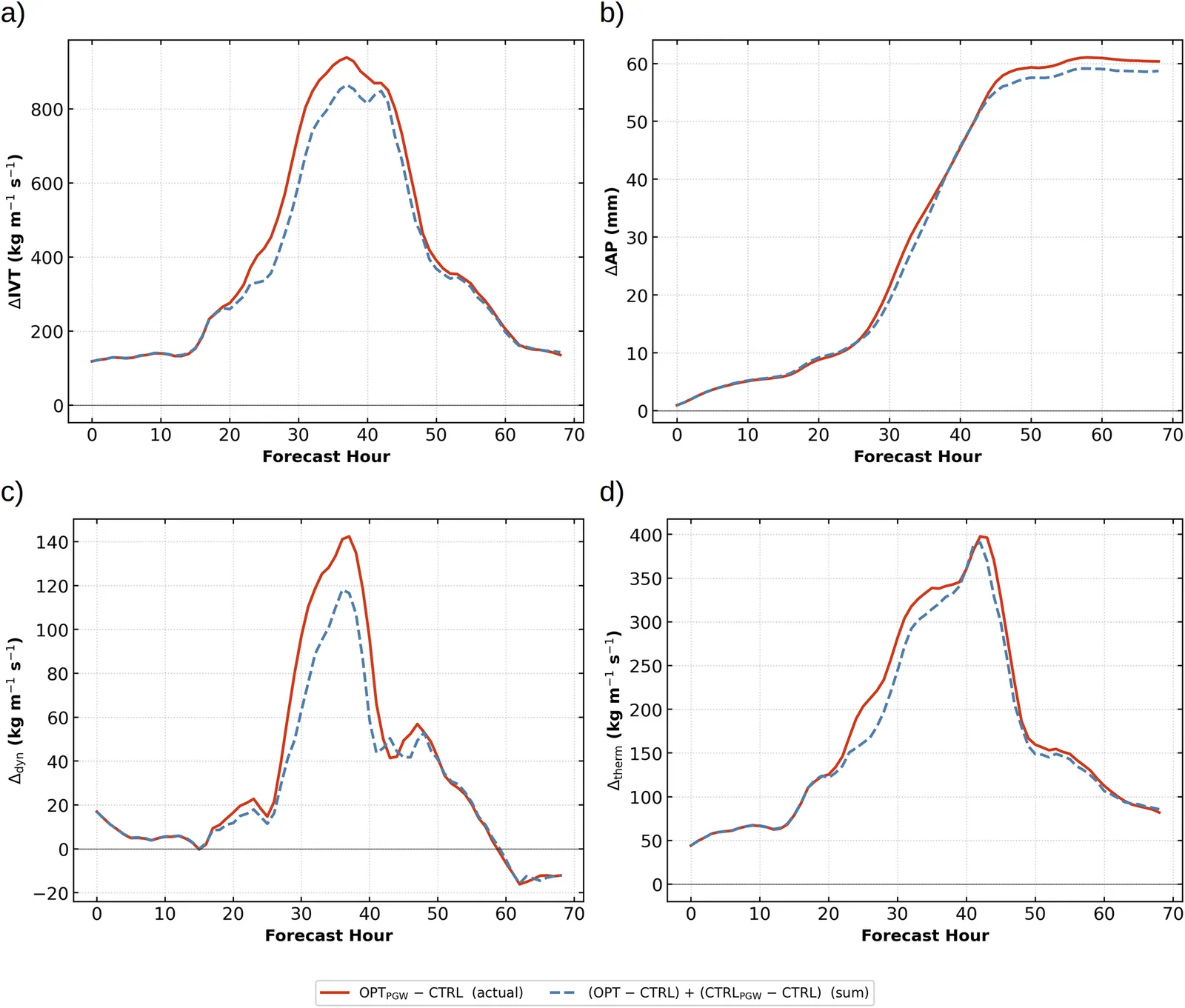
Additivity test for the 2007 event. The solid red line shows the actual combined response (OPT_PGW_−CTRL), and the dashed blue line shows the direct sum of the individual perturbation responses [(OPT–CTRL) + (CTRL_PGW_–CTRL)]. **a** *Δ*IVT (kg m^−1^ s^−1^), **b** *Δ*AP (mm), **c** *Δ*_dyn_ (kg m^−1^ s^−1^), and **d** *Δ*_therm_ (kg m^−1^ s^−1^).

The contribution of non-linear interactions arising from the combined OPT and PGW perturbations is assessed by comparing the IVT obtained from the linear sum of the separate OPT and PGW experiments with the IVT produced by the full, combined experiment. Figure 9 shows results for the 2007 event and confirms that to a large extent, the actual combined response (OPT_PGW_–CTRL) is very close to the direct sum of the individual perturbations. Both *Δ*IVT curves peak near 600 kg m^−1^ s^−1^ at hour 40 and track closely throughout the simulation (Fig. 9a), with differences generally below 10% of the combined signal. The *Δ*_dyn_ and *Δ*_therm_ components individually show similar agreement (Fig. 9c, d), with the largest departures appearing in *Δ*AP after hour 50 (Fig. 9b), where the sum reaches ~57 mm compared to ~60 mm for the actual combined response. Results for other events are shown in Supplementary Figs. 3–6.

### Precipitation efficiency

**Fig. 10:**
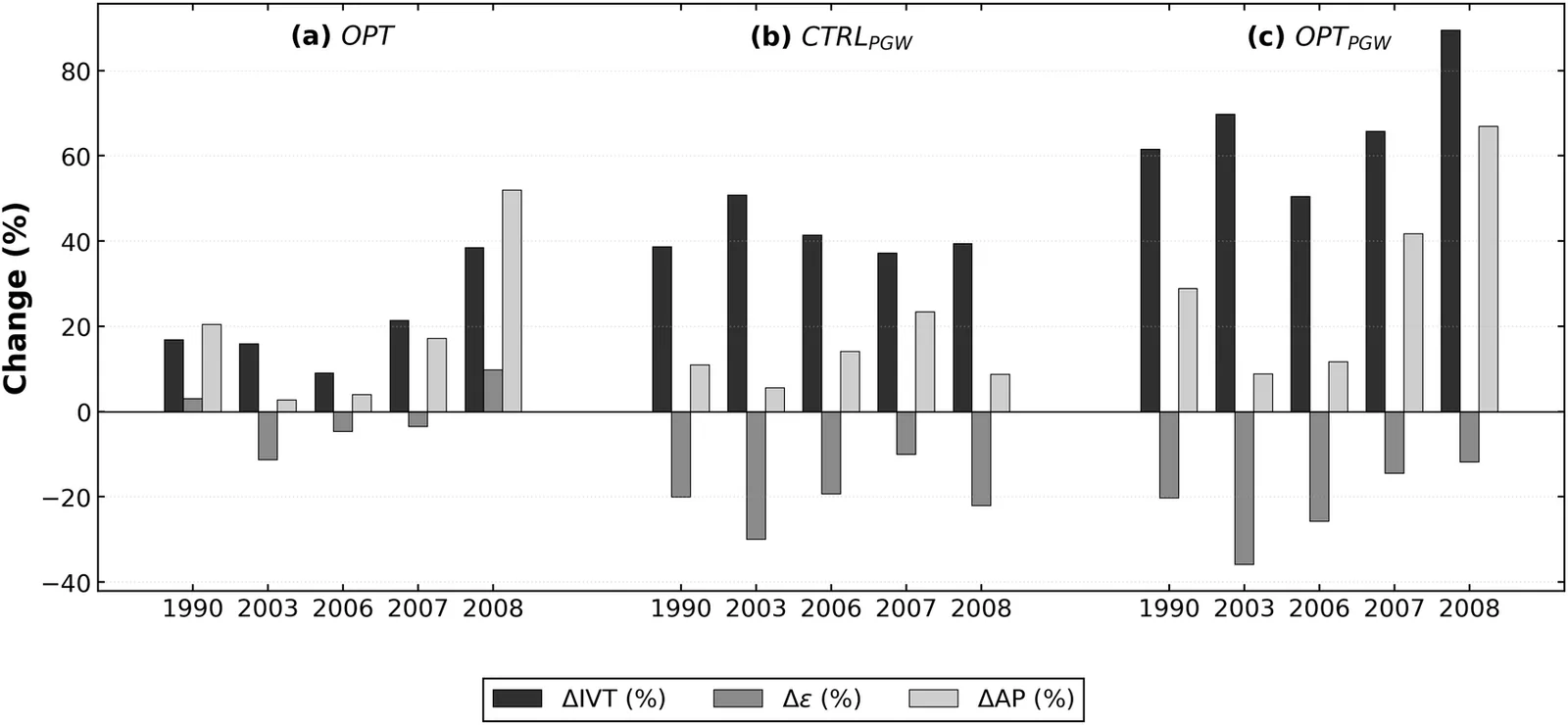
Percentage change in IVT, precipitation efficiency (*ε*), and accumulated precipitation (AP) for each event, evaluated over the 24–54 h forecast window. In each year group, bars show, left to right, *Δ*IVT (black), *Δε* (grey), and *Δ*AP (light grey). **a** OPT–CTRL, **b** CTRL_PGW_–CTRL, and **c** OPT_PGW_–CTRL. The y-axis range is shared across all three panels.

We decompose changes in the accumulated precipitation (*Δ*AP) in terms of changes in IVT and the precipitation efficiency (*Δε*) as defined in Eq. (10) (Fig. 10). Under the optimization (Fig. 10a), *Δ*IVT is positive for all events (9–38%), the efficiency response is comparatively small (−11 to +10%), and *Δ*AP is positive in every case, reaching +52% for the 2008 event. Under PGW (Fig. 10b), *Δ*IVT is again uniformly positive (+37 to +51%), but efficiency decreases sharply for several events, by −29% for 2003, −20% for 1990, and −10 to −22% for 2006–2008, yet *Δ*AP stays positives for all events. In the combined experiment (Fig. 10c), the contrast is sharpened further: the 2008 event, with the largest *Δ*IVT (+89%) and the smallest efficiency loss (−12%), produces *Δ*AP of +67%, while the 2003 event, despite +70% more IVT, loses 36% of its efficiency and ends with +10% precipitation. The equivalent results for the mean maximum precipitation is presented in Supplementary Fig. 7.

### Synoptic situation

**Fig. 11:**
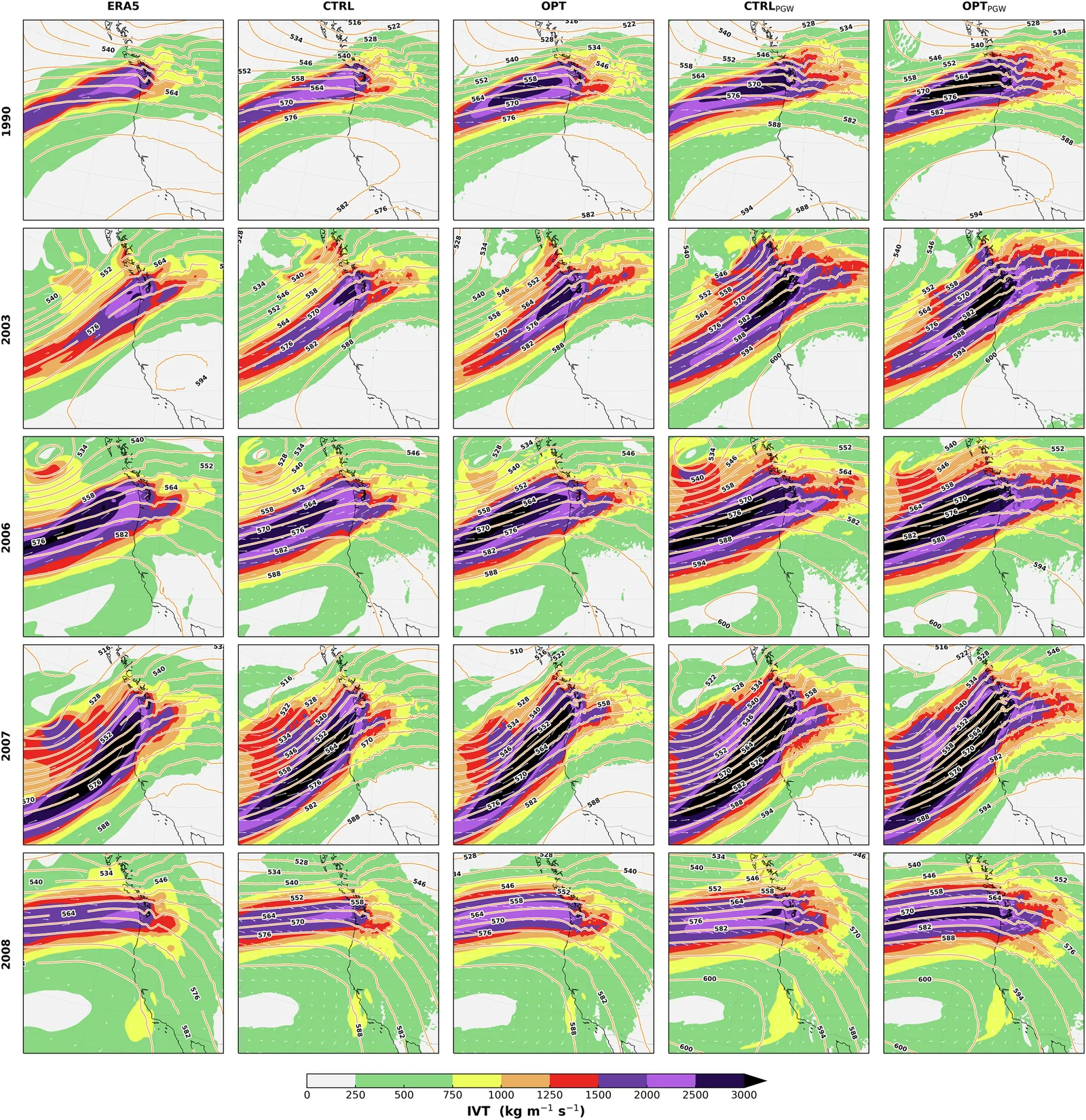
Synoptic structure of each event at peak IVT for all four simulation configurations and ERA5. Rows show the five events (1990, 2003, 2006, 2007, 2008); columns show ERA5, CTRL, OPT, CTRL_PGW_, and OPT_PGW_. Shading: IVT (kg m^−1^ s^−1^). White arrows: 850 hPa winds. Orange contours: 500 hPa geopotential height (dam).

Figure 11 presents the synoptic structure of each event at peak IVT under all four configurations. The CTRL column shows that the model captures the canonical structure of landfalling ARs, with long, narrow filaments of high IVT connecting the subtropical Pacific to the BC coast, embedded within westerly flow at 500 hPa and underpinned by 850 hPa winds aligned with the moisture transport. The events span a range of synoptic configurations, from zonal ARs (1990, 2008) embedded in strong westerly flow to more southwesterly orientations (2003, 2007) associated with deeper troughs over the eastern North Pacific. The 2006 event displays a closed cyclonic circulation to the north of the AR. Comparison with ERA5 (first column) further shows that the model faithfully reproduces the synoptic characteristics of the driving reanalysis.

The OPT column shows that the optimization broadens and intensifies each AR while preserving its synoptic identity. The position and orientation of the AR filaments, the configuration of the 500 hPa height pattern, and the 850 hPa wind direction are all recognizable amplifications of the corresponding CTRL fields rather than qualitatively different states. The IVT incident angles at landfall remain close to their CTRL values across all events and experiments (Supplementary Fig. 1). This supports the storyline interpretation of the framework, where the optimization explores the neighbourhood of each starting event in phase space and produces more intense versions of the same synoptic configuration rather than constructing a new event from scratch.

The CTRL_PGW_ column shows the spatial signature of the PGW perturbation as a broadly intensified IVT field with little change in synoptic structure, consistent with a thermodynamic amplification of the historical event. The OPT_PGW_ column combines both effects, with the most extensive regions of high IVT across all configurations and a synoptic structure that follows the OPT pattern with enhanced moisture content.

## Discussion

**Fig. 12:**
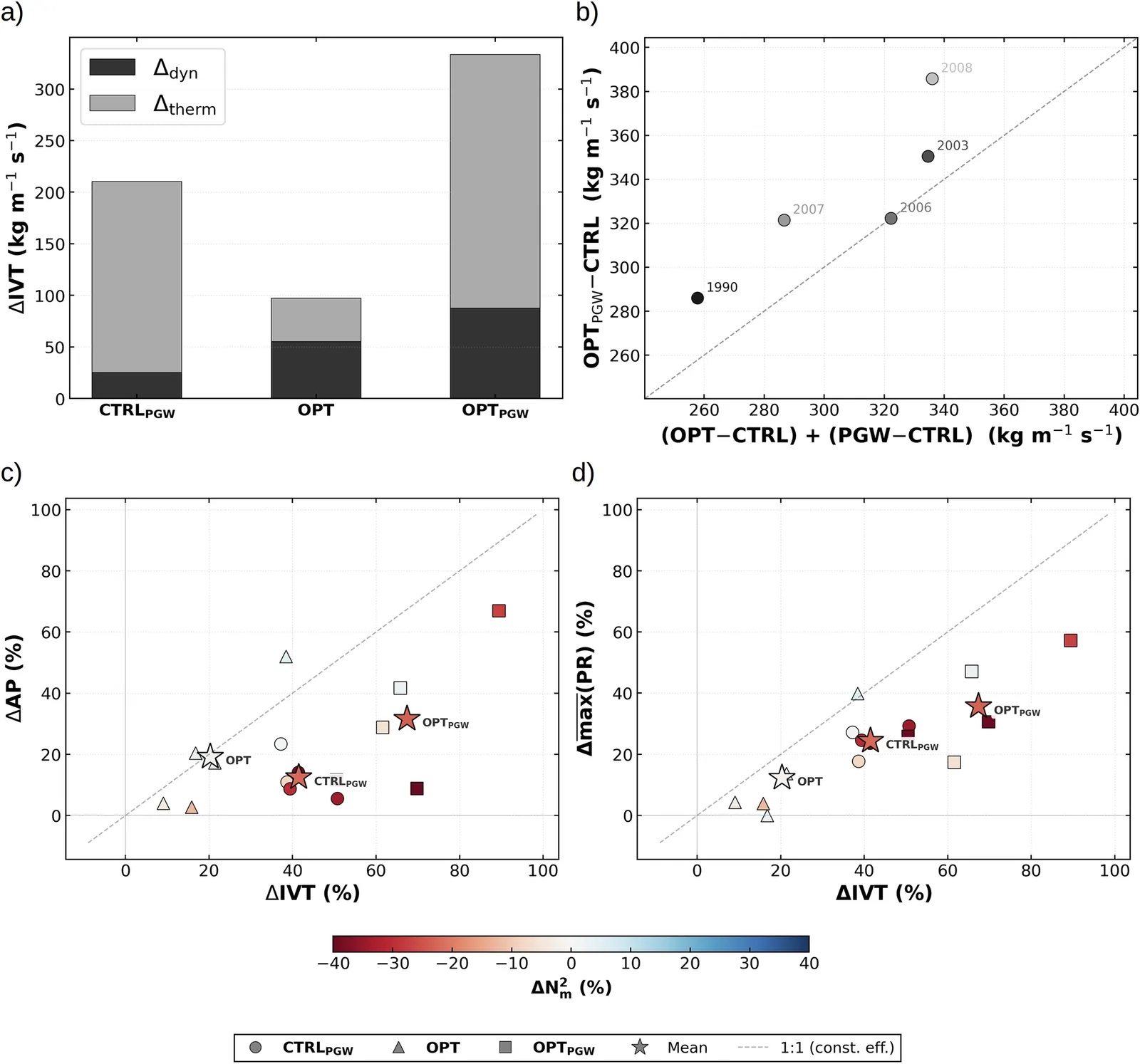
Synthesis across events and experiments. **a** Mean IVT change decomposed into dynamic (*Δ*_dyn_) and thermodynamic (*Δ*_therm_) components, averaged across all five events for each experiment. **b** Actual combined *Δ*IVT (OPT_PGW_–CTRL) vs. the sum of individual responses [(OPT–CTRL) + (CTRL_PGW_–CTRL)] for each event year. The dashed line indicates 1:1. **c** *Δ*AP vs. *Δ*IVT (%) for all 15 event--experiment combinations. Markers are coloured by $\Delta {N}_{m}^{2}$ (%) and shaped by experiment type (triangles: OPT; circles: CTRL_PGW_; squares: OPT_PGW_). The dashed line indicates constant precipitation efficiency. **d** As in panel (**c**), but for the temporal mean of the spatial maximum precipitation rate in place of the domain-averaged accumulated precipitation.

Figure 12 synthesizes the results across all events and experiments. The change in mean IVT (Fig. 12a) shows that in the CTRL_PGW_ experiments, *Δ*_therm_ (184.8 kg m^−1^ s^−1^) is over seven times larger than *Δ*_dyn_ (25.2 kg m^−1^ s^−1^). In the OPT experiment, *Δ*_dyn_ (55.2 kg m^−1^ s^−1^) exceeds *Δ*_therm_ (42.1 kg m^−1^ s^−1^). In the combined experiment (OPT_PGW_), both components contribute to IVT increases, primarily driven by the thermodynamic component, with *Δ*_dyn_ = 87.8 kg m^−1^ s^−1^ and *Δ*_therm_ = 245.6 kg m^−1^ s^−1^.

The pathway separation above is consistent across events. Dynamics dominate OPT, moisture dominates PGW, additivity holds within 15%, and precipitation efficiency drops under PGW but not under OPT, in all five events. Since these are the most intense ARs in the historical record over the region, the decomposition describes how the most extreme historical ARs amplify, not a representative sample.

The spatial fields in Fig. 11 support the plausibility of the amplified states, with the OPT and OPT_PGW_ columns showing recognizable amplifications of the CTRL synoptic configuration rather than qualitatively different patterns. The optimization produces consistent IVT amplification across all five events, with the *Δ*IVT decomposition showing that this amplification is predominantly driven by changes in the dynamical term during the core phase of the event, with the thermodynamic component contributing to increases after the core phase. This result likely follows from the strong constraints imposed on the thermodynamic state of the control events. Because the selected ARs occur during the fall and early winter and are already near column saturation (Fig. 1c), the atmosphere has limited remaining capacity to increase its moisture load at the prevailing temperatures. Consequently, the optimization primarily acts on the component of IVT that remains less constrained—the wind field—to achieve further intensification. The temperature increases observed in the optimized simulations (Fig. 3d) provide an additional thermodynamic pathway to increases in IVT. Since relative humidity remains near saturation in both the control and optimized runs, a warmer atmosphere at saturation holds more moisture in absolute terms. The 1–3 °C warming within the optimized AR corridor thus increases the water vapor content. The optimization therefore amplifies IVT through two complementary mechanisms: a direct increase in wind-driven moisture flux, and an indirect increase in moisture content via warmer temperatures at saturation.

The simulations driven using the PGW method also show consistent increases in *Δ*IVT magnitudes across all events (Fig. 12a). This implies that the dynamical pathways explored using the OPT simulations here can produce IVT increases of approaching magnitude to those expected from end-of-century warming, but under present-day climate conditions. For these events, intensities comparable to those obtained under end-of-century warming are reached through dynamical restructuring alone, without any increase in global mean temperature. These results raise the possibility that thermodynamic scaling of historical events may not by itself capture the range of physically plausible AR amplification, although establishing this beyond the present set of storylines would require analysis of a wider range of cases.

The additivity scatter (Fig. 12b) compares the actual combined *Δ*IVT (OPT_PGW_–CTRL) with the sum of the individual responses [(OPT–CTRL) + (CTRL_PGW_–CTRL)] for each event year. All five events fall close to the 1:1 line, consistent with the decomposition showing that the dynamical and thermodynamic pathways act on largely independent components of the IVT budget. However, for all events but one, the combined response slightly exceeds the linear sum, indicating a modest but systematic positive non-linear effect. This is consistent with the result noted in the combined perturbation results: the optimized circulation transports the moister PGW air more efficiently toward the coast, producing a thermodynamic contribution (*Δ*_therm_) in the combined experiment that peaks at 610–931 kg m^−1^ s^−1^ (Fig. 8b), exceeding the CTRL_PGW_ values of 390–585 kg m^−1^ s^−1^ (Fig. 6b). Within these five storylines, the most amplified events arise when both dynamical and thermodynamic perturbations are applied together. Since these events are among the most intense historical ARs in the region, the amplified storylines lie above the current empirical upper tail, suggesting that this tail is shaped by the compounding of dynamical and thermodynamic drivers rather than by either pathway alone.

This contrast also illustrates the limitation of selecting events by IVT. The 2003 event has the highest IVT in the set but produces relatively little precipitation under amplification because of its sharp efficiency loss, while the 2008 event has lower IVT but converts that moisture transport into precipitation more effectively. Events with high precipitation efficiency at moderate IVT would not be captured by an IVT-based ranking, and the present analysis does not include such events. The pathways characterized here therefore describe how high-IVT extreme events can be amplified, and additional work using a precipitation-aware selection or optimization target would be needed to extend the analysis to events whose impact stems primarily from efficient conversion of moisture transport to precipitation.

Finally, the *Δ*AP vs. *Δ*IVT scatter plot (Fig. 12c) for the relative changes (in %) includes results for all 15 experiments. Despite producing IVT increases, the two amplification pathways differ in their downstream precipitation response. Under the optimization, precipitation efficiency changes are small (−11 to +10%; Fig. 10a), and the OPT experiments (triangles) cluster near or above the 1:1 reference line, with *Δ*IVT values of 9–38% and *Δ*AP values of 2–52%. Under the PGW method, precipitation efficiency decreases sharply, ranging from −10 to −29% (Fig. 10b): the CTRL_PGW_ (circles) and OPT_PGW_ (squares) experiments span a wider range of *Δ*IVT (37–89%) and *Δ*AP (7–67%), but all fall well below the 1:1 line. The efficiency losses are systematically linked to reductions in the moist static stability ${N}_{m}^{2}$ in the 900–500 hPa layer, with most PGW experiments showing $\Delta {N}_{m}^{2}$ values below −15%. The contrast between the 2003 and 2008 events under the combined experiment (Fig. 10c) illustrates the range of outcomes: the 2003 event, despite a +70% increase in IVT, loses 36% of its efficiency and produces only +10% more precipitation, while the 2008 event, with the largest *Δ*IVT (+89%) and the smallest efficiency loss (−12%), produces +67% more precipitation. Figure 12d presents the same analysis but for the temporal mean of the spatial maximum precipitation rate rather than the domain-averaged accumulated total. The individual events cluster more tightly around the 1:1 line, and the mean efficiency losses for the CTRL_PGW_ and OPT_PGW_ experiments are reduced compared to panel (c), indicating that the stability-driven efficiency losses have a smaller effect on peak precipitation rates than on domain-averaged totals.

A plausible explanation is that under Clausius–Clapeyron scaling, the equivalent potential temperature *θ*_*e*_ increases more near the surface, where moisture has increased most, than higher up in the column. The vertical gradient ∂*θ*_*e*_/∂*z* therefore weakens, accounting for the ${N}_{m}^{2}$ decrease noted above. In a more stable column, air forced over the mountains rises sharply, cools quickly, and rains out most of its moisture before crossing the barrier. As stability decreases, the ascent is gentler and spread over a wider area, so less of the moisture is lost on the way up. This offers a mechanism for why the CTRL_PGW_ and OPT_PGW_ experiments fall below the 1:1 line in Fig. 12c despite their large *Δ*IVT. This seems to hold for all these events and the ones with the lowest efficiency are the ones with the largest decrease in stability.

This study combined a differentiable hybrid global climate model (NGCM) with a high-resolution regional climate model (CRCM6-GEM5) to construct physically plausible storylines of extreme ARs making landfall along the coast of British Columbia. By formulating event generation as an optimal control problem, we identified small perturbations to historical initial conditions that maximise IVT at landfall, and compared the resulting dynamical amplification against thermodynamic amplification of the events in current and future climates. Three principal findings emerge.

First, the optimization reveals that dynamical restructuring of the wind field can amplify IVT by magnitudes comparable to those expected from end-of-century thermodynamic warming under SSP5–8.5. Across the five events examined, the dynamical perturbation produced mean IVT increases dominated by the dynamic component (*Δ*_dyn_ = 55.2 kg m^−1^ s^−1^), while the PGW signal produced increases of similar magnitude dominated almost entirely by the thermodynamic component (*Δ*_therm_ = 184.8 kg m^−1^ s^−1^). This demonstrates that in these events, extremes as those projected for a warmer climate are physically accessible today through rare but plausible configurations of the large-scale circulation, without any increase in global mean temperature.

Second, the two amplification pathways are largely independent and nearly additive. When applied simultaneously, the combined *Δ*IVT closely matches the sum of the individual responses, with differences generally below 15%. The combined experiment also reveals a positive non-linear interaction: the optimized circulation transports the moister PGW air more efficiently toward the coast, producing a thermodynamic contribution that exceeds the PGW-only values. Since the events analysed here are among the most intense historical ARs in the region, this result suggests that the most extreme physically plausible ARs arise from the compounding of dynamical and thermodynamic drivers, and that neglecting either pathway risks underestimating the upper tail of AR intensity.

Third, the dynamical and thermodynamic pathways differ in the resulting precipitation. The dynamical perturbation increases both IVT and accumulated precipitation for all events, with precipitation efficiency remaining largely unchanged (−11 to +10%). Under PGW, however, a reduction in precipitation efficiency by up to 29% is noticed.

These results carry several implications for the assessment of AR-related risk. The existence of unobserved dynamical pathways to extreme intensity suggests that thermodynamic scaling of the historical record, while necessary, is insufficient to fully characterize the envelope of physically plausible events. Storyline-based approaches that also explore dynamical variability, such as the framework presented here, can complement conventional large-ensemble and statistical methods to better constrain the upper bounds of AR hazard.

Several limitations should be noted. The optimization was performed on the global model and not propagated through the regional model, meaning that the perturbations identified as optimal at the global scale may not remain optimal once resolved at high resolution with full physics. Sea surface temperatures were held fixed, likely constraining the range of achievable moisture amplification. Future work employing end-to-end differentiable regional models (e.g., ref. ^42^) or storm-resolving global models (e.g., ref. ^43^) could address these limitations and further refine the physical upper bounds of extreme AR intensity.

## Methods

Our framework couples a differentiable global model with high-resolution regional downscaling and PGW perturbations. First, we select extreme AR events based on IVT intensity over the region of study from the EDARA catalogue^41^. Second, we use the selected events to initialize the NGCM differentiable global climate model^44^ at 1.4° horizontal resolution. An optimal control problem that identifies the smallest perturbation to atmospheric initial conditions capable of maximising AR intensity over a coastal target domain is then solved. NGCM is fully differentiable, so the gradient of the objective with respect to the initial state can be computed exactly via backpropagation, making the optimization computationally tractable. Third, the resulting global fields, both control and optimized, are dynamically downscaled with the CRCM6-GEM5 regional climate model at 0.11° (~12 km) grid spacing to resolve the orographic interactions that govern precipitation at landfall. In the final step, PGW perturbations are applied to the lateral boundary conditions of selected simulations, superimposing a thermodynamic climate change signal while leaving the large-scale circulation unmodified.

### AR intensity metric and event selection

We quantify AR intensity using IVT, defined as the magnitude of the column-integrated horizontal moisture flux:

1 $${\rm{IVT}}(\phi ,\theta ,t)=\sqrt{{Q}_{u}^{2}+{Q}_{v}^{2}},$$

where

2 $${Q}_{u}=\frac{1}{g}\int\,q\,u\,{\rm{d}}p\,,\qquad {Q}_{v}=\frac{1}{g}\int\,q\,v\,{\rm{d}}p\,,$$

with specific humidity *q*, zonal and meridional wind components *u* and *v*, pressure *p*, gravitational acceleration *g*, *ϕ*, and *θ* being the longitude and latitude^45^. Although Integrated vapor Kinetic Energy has been proposed as an alternative^46^, we retain IVT for consistency with most current algorithms used to identify ARs^41,47^. One could also quantify AR intensity using precipitation, which is where the societal impact of ARs is felt, but the NGCM precipitation output is not reliable at the resolution used here, and the only checkpoint with precipitation is the stochastic version of the model, which is not suited to the deterministic framework used in this work.

**Fig. 13:**
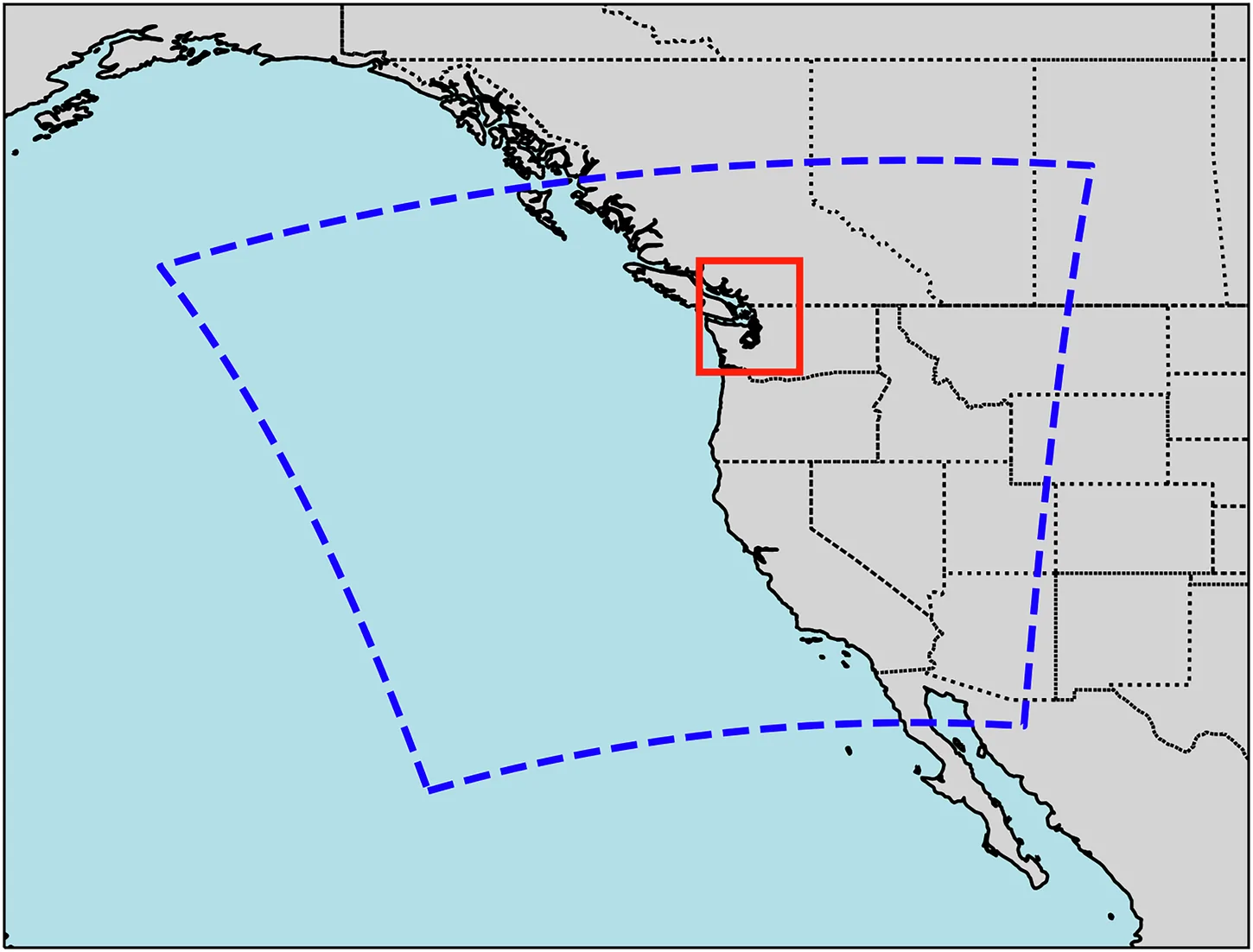
Map showing the simulation domain with dashed blue line and target region with red box.

AR events are drawn from the EDARA catalogue^41^, a global AR inventory derived from the ERA5 reanalysis^48^ that comprises 12 meteorological variables spanning the full reanalysis period with 0.25° resolution. In particular we use the *Q*_*u*_, *Q*_*v*_ components to compute the IVT over the years 1970–2024. We select events in two stages. First, we retain only AR tracks whose footprint intersects a coastal target domain centred over Vancouver, British Columbia (red box in Fig. 13, following^6^). This intersection is established by identifying tracks that spent at least one hour in the targeted region. Second, we rank the retained events by their spatiotemporal mean IVT (Eq. (1)) and select the five most intense cases (Table 1). The five-event sample is set by computational cost rather than by methodological limits. Scaling the analysis to additional events is straightforward but requires a proportional increase in compute time.

**Table 1:** CRCM6-GEM5 simulation matrix of extreme AR events

Simulation	Initialisation date	*N*_opt_	PGW
*Control (CTRL)*			
1990-CTRL	1990-11-23	—	—
2003-CTRL	2003-10-19	—	—
2006-CTRL	2006-11-05	—	—
2007-CTRL	2007-12-02	—	—
2008-CTRL	2008-11-11	—	—
*optimized (OPT)*			
1990-OPT	1990-11-23	150	—
2003-OPT	2003-10-19	150	—
2006-OPT	2006-11-05	150	—
2007-OPT	2007-12-02	150	—
2008-OPT	2008-11-11	150	—
*Control* + *PGW* (CTRL_PGW_)			
1990-CTRL-PGW	1990-11-23	—	*✓*
2003-CTRL-PGW	2003-10-19	—	*✓*
2006-CTRL-PGW	2006-11-05	—	*✓*
2007-CTRL-PGW	2007-12-02	—	*✓*
2008-CTRL-PGW	2008-11-11	—	*✓*
*optimized* + *PGW* (OPT_PGW_)			
1990-OPT-PGW	1990-11-23	150	*✓*
2003-OPT-PGW	2003-10-19	150	*✓*
2006-OPT-PGW	2006-11-05	150	*✓*
2007-OPT-PGW	2007-12-02	150	*✓*
2008-OPT-PGW	2008-11-11	150	*✓*

Each of the five AR events is run under four configurations, each run covering a 70-h period. *N*_opt_ denotes the number of gradient-descent iterations used during the optimization; PGW indicates whether the PGW perturbation was applied to the lateral and surface boundary conditions.

### Optimal perturbation of initial conditions

The optimization of the spatiotemporal-mean IVT of selected ARs is performed using NGCM at 1.4° horizontal resolution and 32 evenly spaced vertical levels on sigma coordinates using the deterministic version of the model^44^. We formulate the generation of unprecedented, extreme AR storylines as an optimal control problem. Let **x**_0_ denote the full atmospheric state at the forecast initialisation time and *Δ***x**_0_ a perturbation applied to that state. We seek the perturbation *Δ***x**_0_ that maximises AR intensity at landfall while remaining as small as possible relative to the model’s own internal variability, by minimising the cost functional

3 $${\mathcal{L}}(\Delta {{\bf{x}}}_{0})=\,\frac{{{\rm{IVT}}}_{{\rm{ref}}}}{\overline{{\rm{IVT}}}}\,+\,\Delta {{\bf{x}}}_{0}^{\top }\,{{\bf{B}}}^{-1}\,\Delta {{\bf{x}}}_{0}\,,$$

where

4 $$\overline{{\rm{IVT}}}=\frac{1}{\tau \,| {\mathcal{D}}| }\mathop{\int}\nolimits_{0}^{\tau }{\iint }_{{\mathcal{D}}}{\rm{IVT}}(\phi ,\theta ,t)\,{\rm{d}}\phi \,{\rm{d}}\theta \,{\rm{d}}t$$

is the IVT averaged over the target domain ${\mathcal{D}}$ and the forecast window *τ* (24–54 h). The first term in Eq. (3) drives the solution toward more intense ARs; the second term penalizes departures from the control state via the background-error covariance matrix **B**, whose diagonal elements are estimated from the variance of the noise used by the NGCM’s stochastic model. This is computed by computing the variance of a 75 member ensemble from NGCM. The optimization method follows closely the work done in ref. ^37^.

Gradients of ${\mathcal{L}}$ with respect to *Δ***x**_0_ are computed by backpropagating through the full NGCM forecast trajectory. The perturbation is then updated iteratively using the Adam optimizer^49^ for *N*_opt_ = 150 iterations per event. For the experiments presented here we use a learning rate of 10^−10^.

### Dynamical downscaling using CRCM6-GEM5

Global fields from both the control and optimized NGCM forecasts are dynamically downscaled using the CRCM6-GEM5 regional climate model^50–53^. The atmospheric component of CRCM6-GEM5 is the Global Environmental Multiscale (GEM) model, version 5^54–56^, developed by the Recherche en Prévision Numérique group at Environment and Climate Change Canada.

**Fig. 14:**
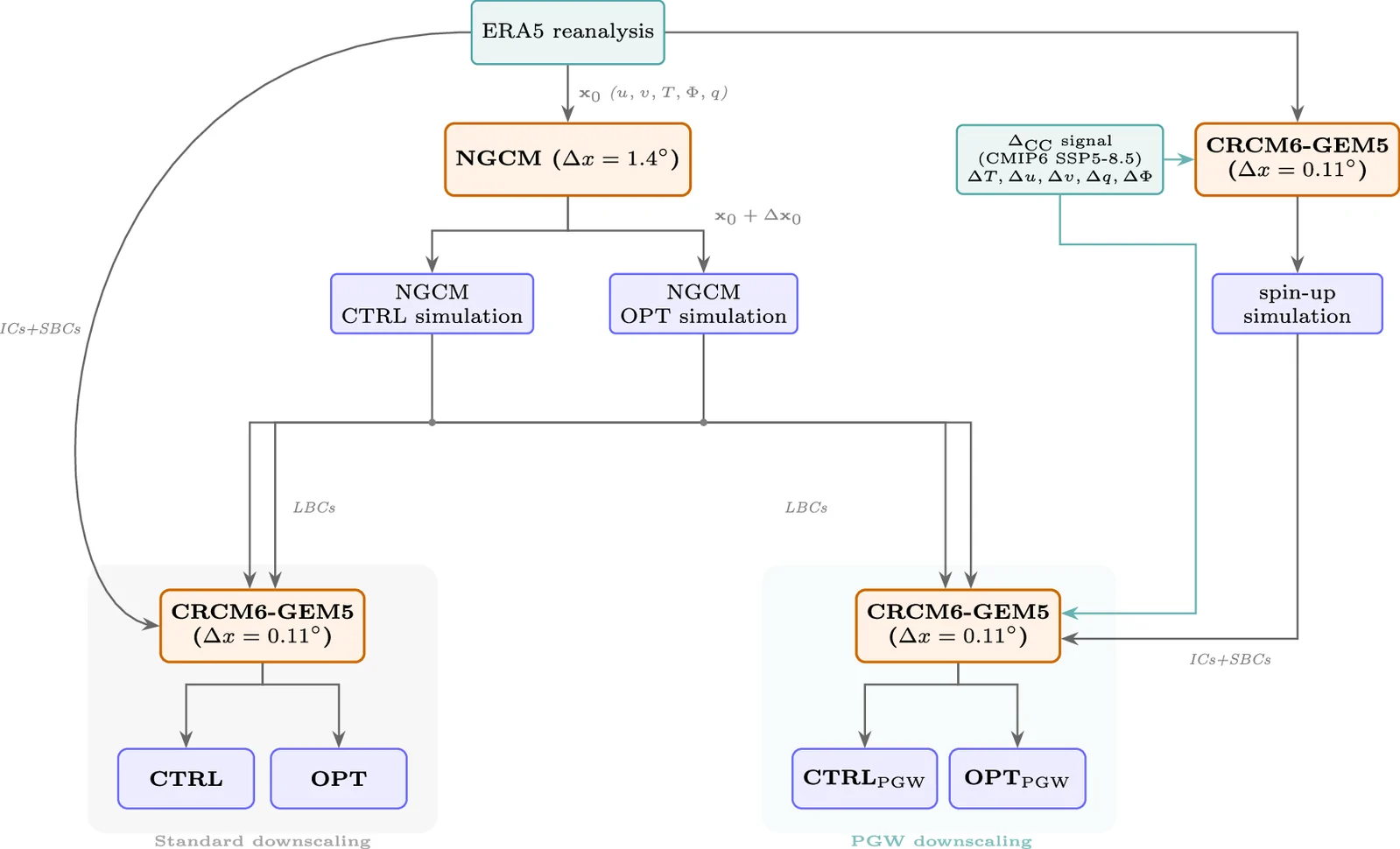
Simulation workflow. ERA5 reanalysis initialises the NGCM model, which produces control and optimized simulations. These are downscaled using the CRCM6-GEM5 model under two configurations: standard (with remaining boundary conditions from ERA5) and PGW (with remaining boundary conditions from an ERA5 spin-up simulation and the CMIP6 SSP5–8.5 climate change signal added). LBCs stands for lateral boundary conditions, ICs for initial conditions, and SBCs for surface boundary conditions.

The model is configured on a 0.11° (~12 km) horizontal grid with 71 hybrid vertical levels extending from ~10 m above the surface to a model top at 10 hPa. The simulation domain is shown in Fig. 13 (dashed blue line). The NGCM provides lateral boundary forcing of winds, temperature, specific humidity, and geopotential, while the remaining surface and boundary fields are taken from ERA5 reanalysis (see the left branch of the diagram in Fig. 14). Lateral boundary conditions from the NGCM are updated hourly.

The physics suite includes the Bechtold shallow-convection scheme^57^, the Kain–Fritsch deep-convection scheme^58,59^ with an elevated-convection extension^60^, a 1.5-order turbulent kinetic energy closure for boundary-layer turbulence^56,61,62^, and the Predicted Particle Properties (P3) microphysics scheme^63,64^, coupled with a subgrid cloud-fraction parameterisation^65,66^. Land surface processes are represented by the Canadian Land Surface Scheme (CLASS) v3.6^67,68^, and lake thermodynamics by the FLake model^69^.

### Pseudo-global warming method

To generate storylines of future AR intensification, we apply the PGW method^27,70,71^ to produce a new set of simulations for all selected events (Table 1). The PGW method consists of superimposing a monthly-mean climate change signal (*Δ*_CC_) on the atmospheric lateral boundary fields (temperature (*T*), horizontal winds (*u*, *v*), specific humidity (*q*) and geopotential height (*Φ*)), and on the surface boundary conditions (sea surface temperatures, SSTs). This climate change signal is computed as the difference between a future projection and the corresponding historical baseline, derived from monthly mean fields averaged across an ensemble of 30 General Circulation Models (GCMs) from the sixth Coupled Model Intercomparison Project (CMIP6). Specifically, *Δ*_CC_ is calculated between the periods 1990–2014 and 2076–2100 under the SSP5–8.5 scenario^72,73^. The full list of GCMs and realizations is provided in Supplementary Table 1.

For the PGW simulations, the land surface is equilibrated through a CRCM6-GEM5 spin-up driven by ERA5 boundary conditions with the *Δ*_CC_ signal applied. The 2003, 2006, 2007, and 2008 events are drawn from a continuous PGW simulation spanning 2000–2024 and are therefore preceded by several years of spin-up^72^. The 1990 event falls outside this period and was run separately with a dedicated one-year spin-up prior to the event. All events thus include at least a one-year spin-up, which is sufficient for land-surface equilibration in PGW applications. Each AR event is then simulated using the CRCM6-GEM5 model with lateral boundary conditions derived from the NGCM simulation with the *Δ*_CC_ signal superimposed, while the surface boundary conditions are initialized from the PGW SSTs and the spun-up land-surface state (see right branch of the workflow diagram in Fig. 14).

### AR event simulations

The modelling chain produces four simulation types for each of the five AR events, yielding 20 simulations in total (Table 1):

- CTRL: CRCM6-GEM5 driven by the control NGCM simulation;
- OPT: CRCM6-GEM5 driven by the optimized NGCM simulation;
- CTRL_PGW_: as CTRL, with the PGW signal added to the lateral and surface boundaries;
- OPT_PGW_: as OPT, with the PGW signal added to the lateral and surface boundaries.

Three difference pairs isolate the effect of each amplification pathway relative to the historical control:

1. OPT-CTRL: effect of the optimization alone;
2. CTRL_PGW_-CTRL: effect of PGW alone (referred to as *CTRL*_*PGW*_);
3. OPT_PGW_-CTRL: combined effect of both perturbations (referred to as *OPT*_*PGW*_).

For each pair, we analyse both the full hourly time series over the 70 h simulation and 30-h time-mean diagnostics evaluated over the 24–54 h window during which IVT is at or near its peak for all five events. To test whether the two pathways interact non-linearly, we also compare the actual combined response (*OPT*_*PGW*_) with the sum of the individual responses [(OPT–CTRL) + (CTRL_PGW_–CTRL)].

### AR events diagnostics

All spatially averaged diagnostics reported are computed from the CRCM6-GEM5 output over a coastal impact region bounded by 46–51°N and 120.5–125°W (red box in Fig. 13).

To attribute the IVT amplification (*Δ*IVT) to changes in the wind field versus changes in moisture content, we decompose *Δ*IVT into dynamical, thermodynamic and residual terms. For a given comparison pair (Section AR event simulations), we denote the control simulation with subscript *c* and the perturbed simulation with subscript *p*, and define the mean state $\overline{(\cdot )}=\frac{1}{2}[{(\cdot )}_{c}+{(\cdot )}_{p}]$. Linearising the moisture-flux components about this mean state gives

5 $$\Delta {Q}_{u}\approx \frac{1}{g}\int\,\bar{q}\,\Delta u\,{\rm{d}}p+\frac{1}{g}\int\,\bar{u}\,\Delta q\,{\rm{d}}p\,,$$

and analogously for *ΔQ*_*v*_, where *Δ*( ⋅ ) = (⋅)_*p*_ − (⋅)_*c*_. This symmetric formulation distributes the wind–humidity covariance equally between the two terms. We project the component changes onto the mean transport direction using the weights ${w}_{u}={\bar{Q}}_{u}/\overline{{\rm{IVT}}}$ and ${w}_{v}={\bar{Q}}_{v}/\overline{{\rm{IVT}}}$, yielding

6 $$\Delta {\rm{IVT}}={\Delta }_{{\rm{dyn}}}+{\Delta }_{{\rm{therm}}}+R\,,$$

where

7 $${\Delta }_{{\rm{dyn}}}={w}_{u}\int\,\bar{q}\,\Delta u\,\frac{{\rm{d}}p}{g}+{w}_{v}\int\,\bar{q}\,\Delta v\,\frac{{\rm{d}}p}{g},$$

8 $${\Delta }_{{\rm{therm}}}={w}_{u}\int\,\bar{u}\,\Delta q\,\frac{{\rm{d}}p}{g}+{w}_{v}\int\,\bar{v}\,\Delta q\,\frac{{\rm{d}}p}{g}\,.$$

The dynamical component (*Δ*_dyn_) represents the contribution of wind changes acting on the mean moisture field, and the thermodynamic component (*Δ*_therm_) represents the contribution of moisture changes advected by the mean winds. The residual *R* = *Δ*IVT − *Δ*_dyn_ − *Δ*_therm_ arises from the linearisation of the IVT magnitude. Across all 15 perturbation experiments, ∣*R*∣ does not exceed 10 kg m^−1^ s^−1^ over the simulation, corresponding to roughly 1% of *Δ*IVT, confirming that the linearisation remains valid even for the most strongly amplified OPT_PGW_ experiments (see Supplementary Fig. 2). Column-mean relative humidity, used as a supporting diagnostic, is computed as the pressure-weighted vertical average of relative humidity across all model levels.

To characterize the atmospheric column’s resistance to orographic lifting, we compute the squared moist Brunt–Väisälä frequency:

9 $${N}_{m}^{2}=\frac{g}{\bar{\theta }}\,\frac{\partial {\theta }_{e}}{\partial z}\,,$$

where $\bar{\theta }$ is the layer-mean potential temperature and *θ*_*e*_ = *θ* + (*L*_*v*_/*c*_*p*_) *q* is the equivalent potential temperature. Here $\theta =T\,{({p}_{0}/p)}^{{R}_{d}/{c}_{p}}$ is the dry potential temperature, *L*_*v*_ = 2.501 × 10^6^ J kg^−1^ the latent heat of vaporization, and *c*_*p*_ = 1004 J kg^−1^ K^−1^ the isobaric specific heat. Using *θ*_*e*_ rather than *θ* accounts for latent-heat release during saturated ascent, making ${N}_{m}^{2}$ the appropriate stability measure under the near-saturated conditions typical of landfalling ARs^74^. Values are computed from time-mean, regional-mean profiles over the 24–54 h simulation window and averaged over the 900–500 hPa orographic layer.

We define precipitation efficiency as the ratio of accumulated precipitation (AP = ∑PR) at the end of the simulation to time-mean IVT over the analysis window:

10 $$\varepsilon =\frac{\sum {\rm{PR}}}{\overline{{\rm{IVT}}}}\,.$$

This metric quantifies how effectively the moisture flux incident on the orographic barrier is converted to surface precipitation. Changes in accumulated precipitation can then be understood as the outcome of a competition between changes in the moisture transport supply (*Δ*IVT) and changes in the precipitation efficiency (*Δε*).

## Supplementary information

Supplementary information

## Data Availability

All data generated using the code and ERA5 data used in this study are openly available from the Copernicus Data Store (https://doi.org/10.24381/cds.143582cf)^48^. The high-resolution simulations will be made available upon request.

## Code availability

https://github.com/timwhittaker/ExtremeStorylines(https://doi.org/10.5281/zenodo.20445765).
