# Supplementary material for: Distinct dynamical and thermodynamic pathways compound to amplify storylines of extreme atmospheric rivers in British Columbia
Source: NPJ Clim Atmos Sci. 2026 Jul 5;9(1):211. doi: 10.1038/s41612-026-01476-8 (PMC13713544; doi:10.1038/s41612-026-01476-8)
Supplement: Supplementary file 1 [file 41612_2026_1476_MOESM1_ESM.pdf]

Supporting Information for Distinct dynamical and thermodynamic pathways compound to amplify storylines of extreme atmospheric rivers in British Columbia

Tim Whittaker[1*] and Alejandro Di Luca[1]

[1]Centre Étude et simulation du climat à l'échelle régionale (ESCER), Département des Sciences de la Terre et de l'Atmosphère, Université du Québec à Montréal, Montréal, Québec, Canada.

*Corresponding author(s). E-mail(s): whittaker.tim@courrier.uqam.ca;

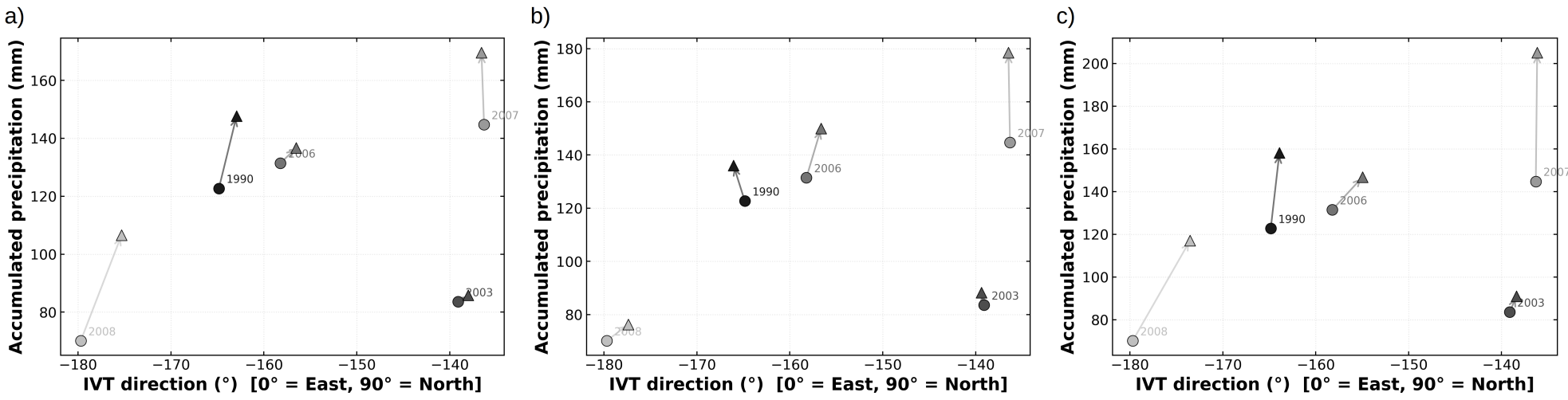

Fig. 1 Accumulated precipitation vs. IVT direction for each event. Circles indicate the CTRL simulation and triangles indicate the perturbed simulation; arrows connect the two for each event. (a) OPT − CTRL, (b) PGW − CTRL, and (c) $\mathrm{OPT_{PGW}}$ − CTRL. Changes in IVT orientation are small relative to the magnitude of the IVT and precipitation anomalies, suggesting that flow direction does not explain the inter-event variability in the precipitation response.

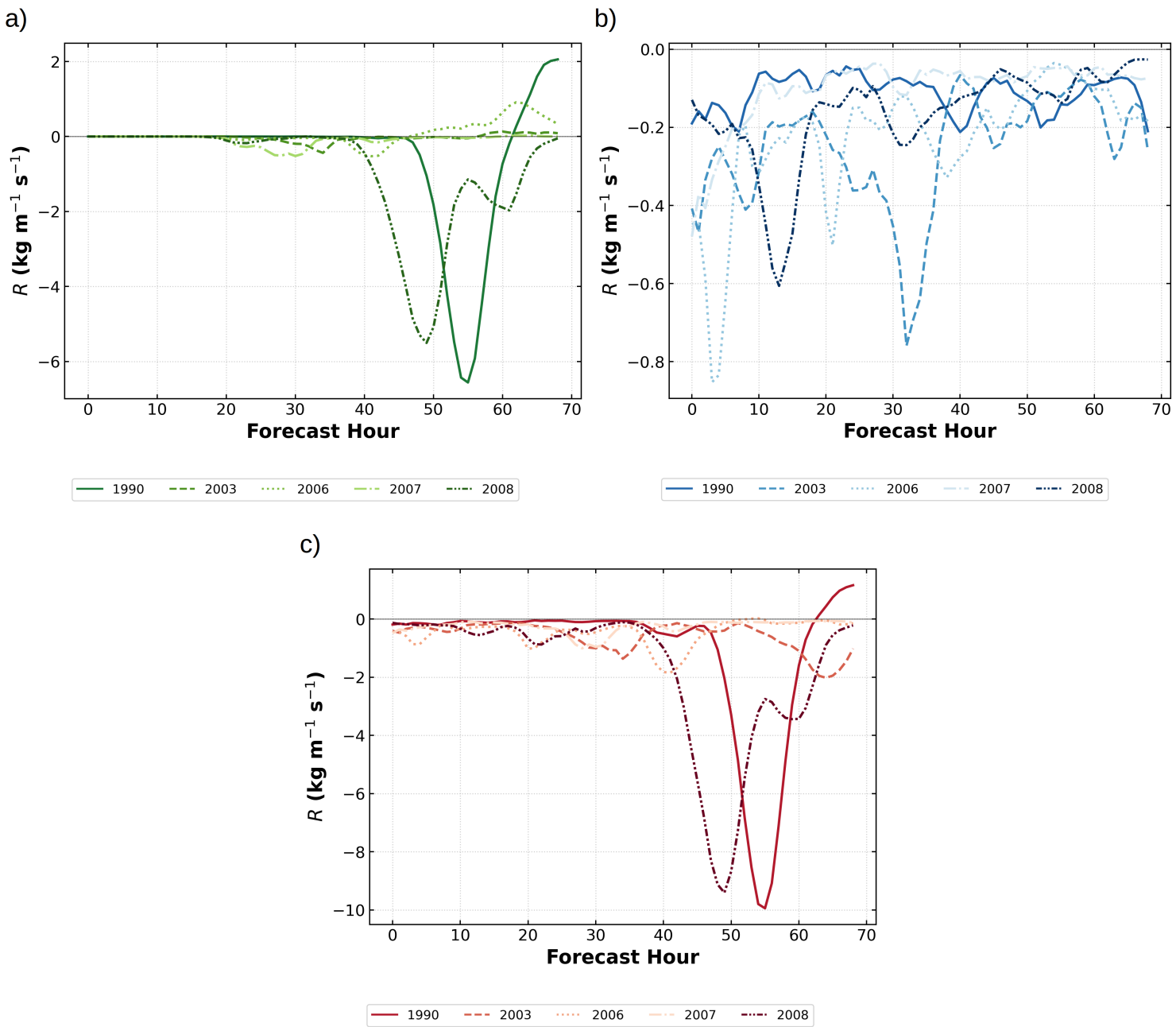

Fig. 2 Residual term of the IVT decomposition for the three difference experiments. (a) OPT − CTRL, (b) PGW − CTRL, and (c) $\mathrm{OPT_{PGW}}$ − CTRL.

1 AR angle dependence

2 Residuals

3 Additivity tests

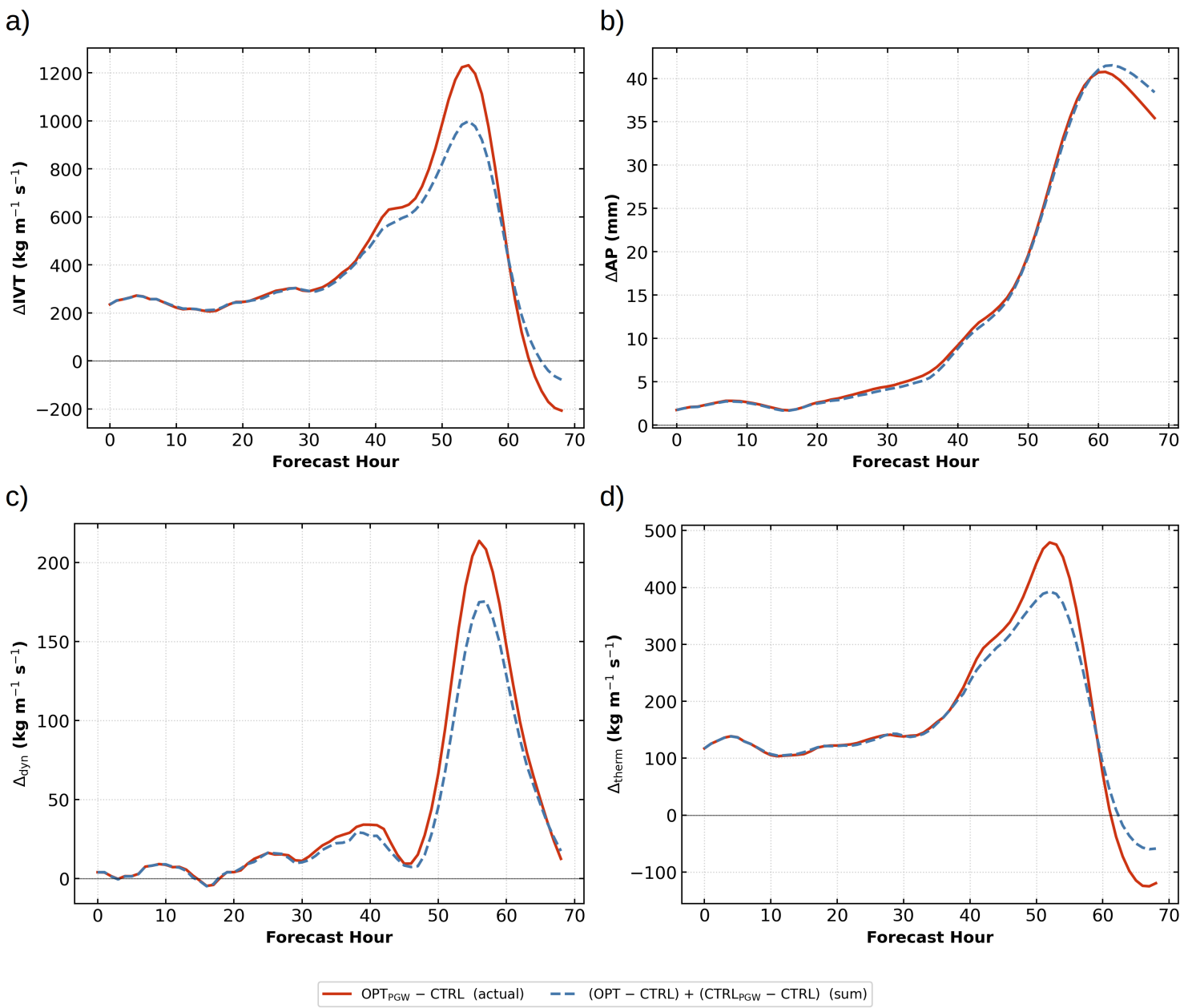

Fig. 3 Additivity test for the 1990 event. The solid red line shows the actual combined response ($\mathrm{OPT_{PGW}} - \mathrm{CTRL}$), and the dashed blue line shows the direct sum of the individual perturbation responses [(OPT − CTRL) + (PGW − CTRL)]. (a) ΔIVT ($\mathrm{kg\,m^{-1}\,s^{-1}}$), (b) ΔAP (mm), (c) Δ_{dyn} ($\mathrm{kg\,m^{-1}\,s^{-1}}$), and (d) Δ_{therm} ($\mathrm{kg\,m^{-1}\,s^{-1}}$).

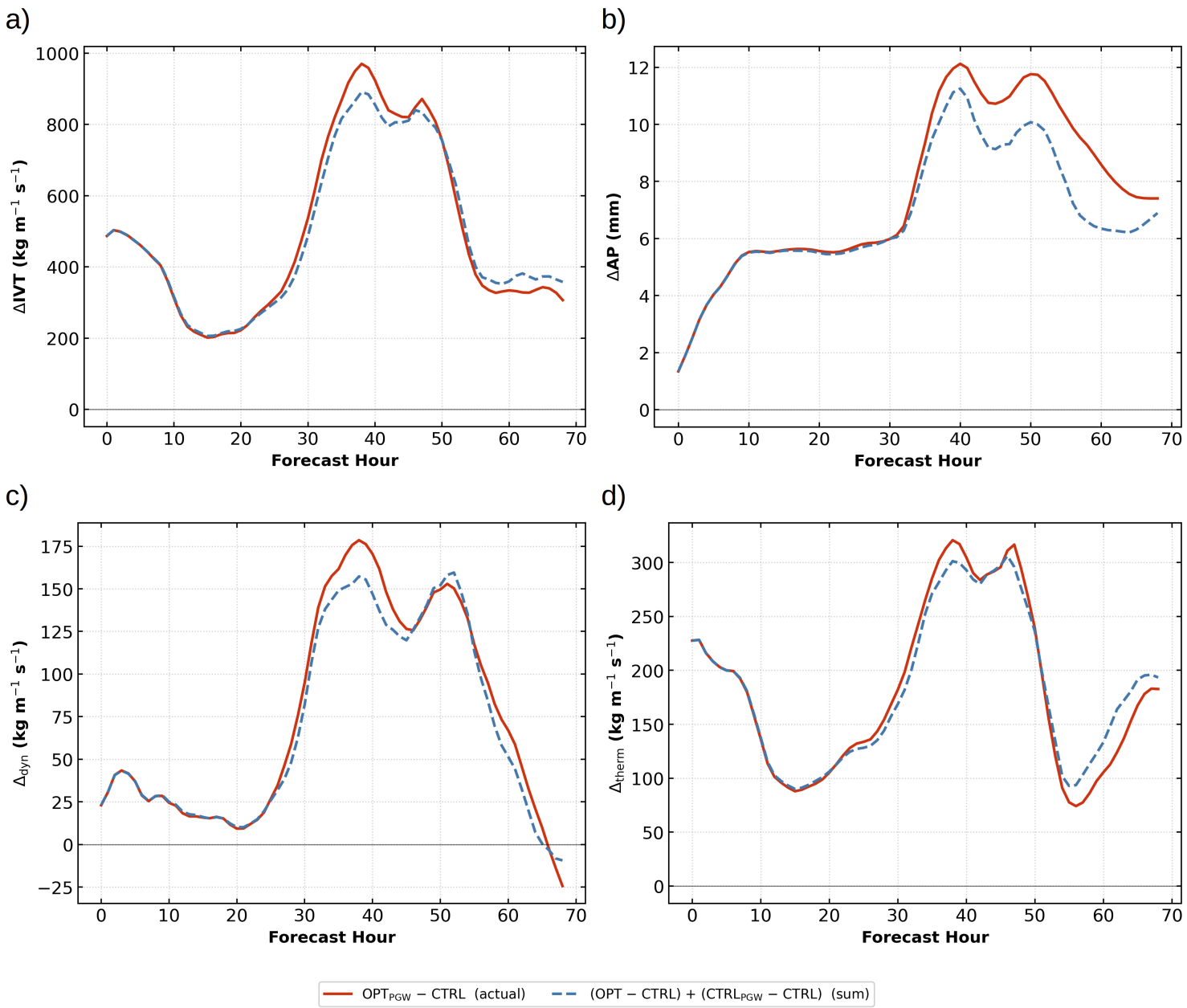

Fig. 4 Additivity test for the 2003 event. The solid red line shows the actual combined response ($\mathrm{OPT_{PGW}} - \mathrm{CTRL}$), and the dashed blue line shows the direct sum of the individual perturbation responses [(OPT − CTRL) + (PGW − CTRL)]. (a) ΔIVT ($\mathrm{kg\,m^{-1}\,s^{-1}}$), (b) ΔAP (mm), (c) Δ_{dyn} ($\mathrm{kg\,m^{-1}\,s^{-1}}$), and (d) Δ_{therm} ($\mathrm{kg\,m^{-1}\,s^{-1}}$).

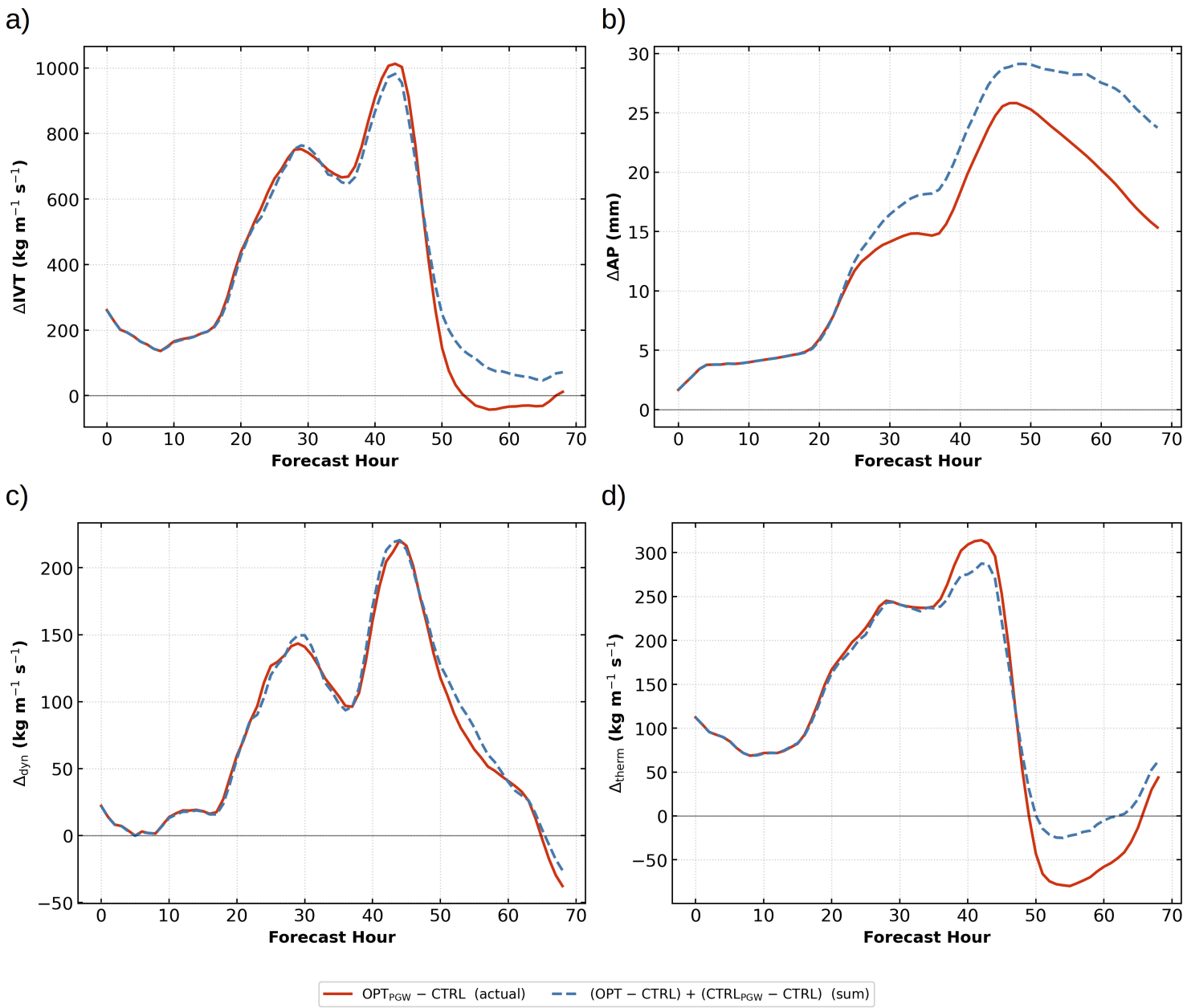

Fig. 5 Additivity test for the 2006 event. The solid red line shows the actual combined response ($OPT_{PGW} - CTRL$), and the dashed blue line shows the direct sum of the individual perturbation responses [(OPT − CTRL) + (PGW − CTRL)]. (a) ΔIVT ($kg\,m^{-1}\,s^{-1}$), (b) ΔAP (mm), (c) Δ_{dyn} ($kg\,m^{-1}\,s^{-1}$), and (d) Δ_{therm} ($kg\,m^{-1}\,s^{-1}$).

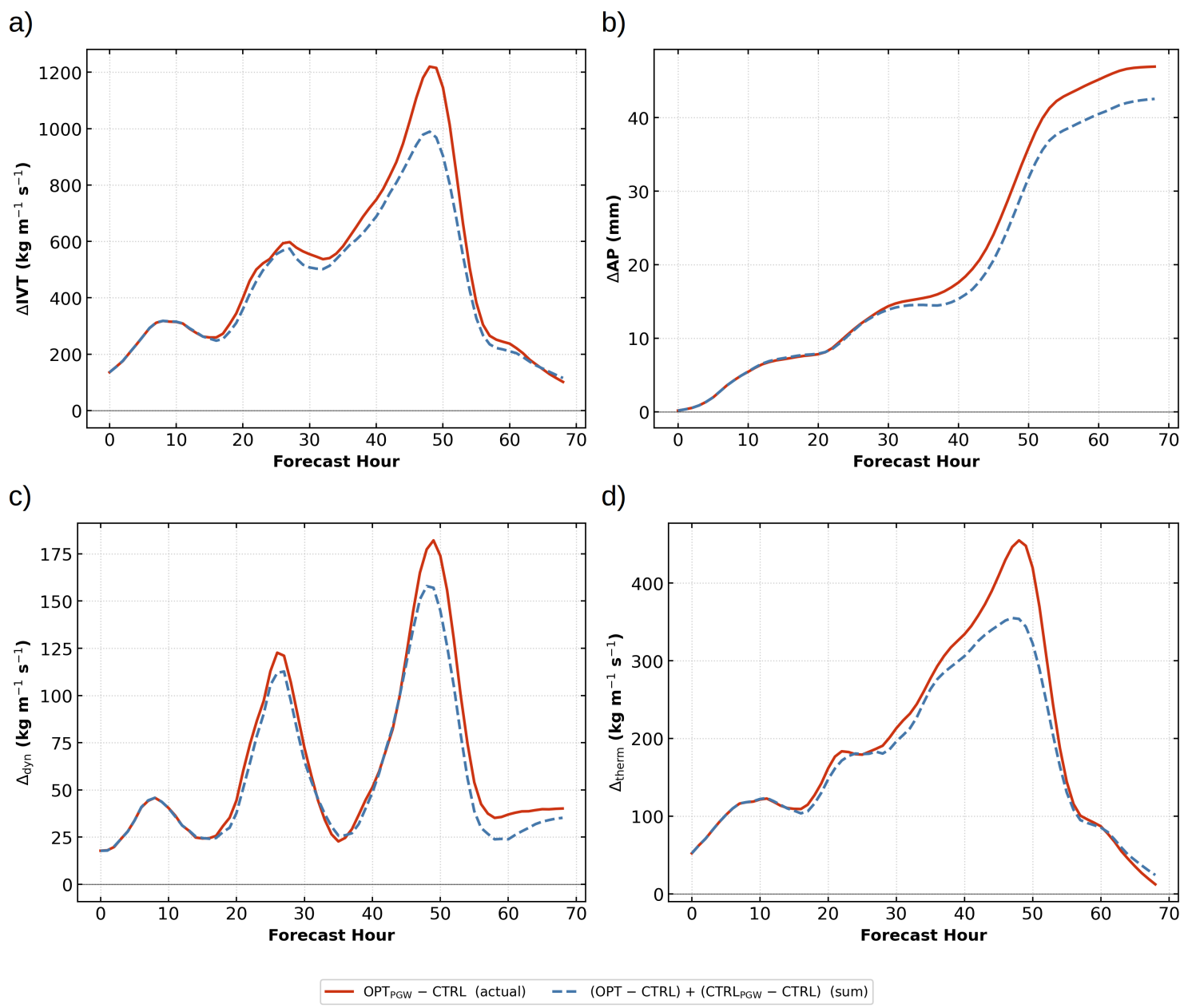

Fig. 6 Additivity test for the 2008 event. The solid red line shows the actual combined response ($\mathrm{OPT_{PGW}} - \mathrm{CTRL}$), and the dashed blue line shows the direct sum of the individual perturbation responses [(OPT − CTRL) + (PGW − CTRL)]. (a) ΔIVT ($\mathrm{kg\,m^{-1}\,s^{-1}}$), (b) ΔAP (mm), (c) Δ_{dyn} ($\mathrm{kg\,m^{-1}\,s^{-1}}$), and (d) Δ_{therm} ($\mathrm{kg\,m^{-1}\,s^{-1}}$).

4 Maximum Precipitation

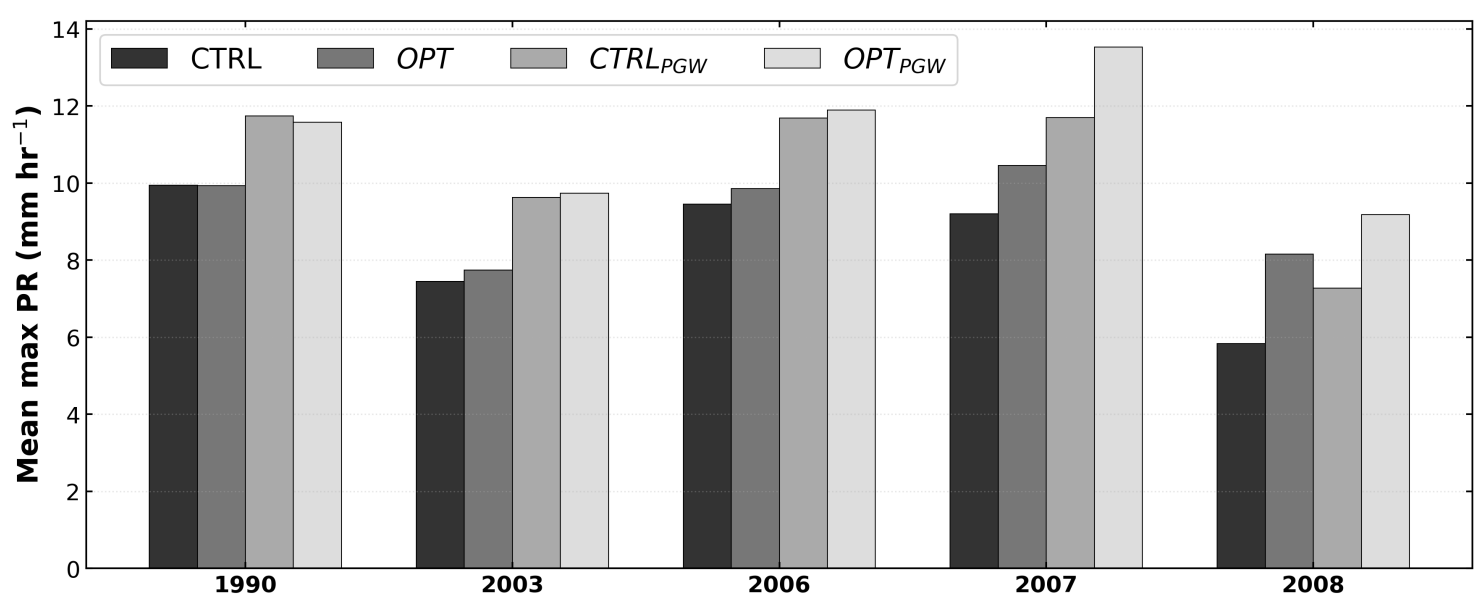

Fig. 7 Temporal mean of the spatial maximum precipitation rate for each event under the CTRL, OPT, $\mathrm{CTRL_{PGW}}$, and $\mathrm{OPT_{PGW}}$ simulations.

5 CMIP6 climate change delta

GCM	Realization
ACCESS-CM2	r1i1p1f1
ACCESS-ESM1-5	r1i1p1f1
CanESM5	r1i1p1f1
CMCC-CM2-SR5	r1i1p1f1
CMCC-ESM2	r1i1p1f1
CNRM-CM6-1	r1i1p1f2
CNRM-CM6-1-HR	r1i1p1f2
CNRM-ESM2-1	r1i1p1f2
EC-Earth3	r1i1p1f1
EC-Earth3-CC	r1i1p1f1
EC-Earth3-Veg	r1i1p1f1
EC-Earth3-Veg-LR	r1i1p1f1
FGOALS-f3-L	r1i1p1f1
GFDL-ESM4	r1i1p1f1
GISS-E2-1-G	r1i1p1f2
GISS-E2-1-H	r1i1p1f2
HadGEM3-GC31-LL	r1i1p1f3
HadGEM3-GC31-MM	r1i1p1f3
INM-CM4-8	r1i1p1f1
INM-CM5-0	r1i1p1f1
IPSL-CM6A-LR	r1i1p1f1
KACE-1-0-G	r1i1p1f1
KIOST-ESM	r1i1p1f1
MCM-UA-1-0	r1i1p1f2
MIROC-ES2L	r1i1p1f2
MIROC6	r1i1p1f1
MPI-ESM1-2-HR	r1i1p1f1
MPI-ESM1-2-LR	r1i1p1f1
MRI-ESM2-0	r1i1p1f1
UKESM1-0-LL	r1i1p1f2

Table 1 List of the 30 CMIP6 General Circulation Models (GCMs) used to compute the climate change delta applied in the PGW simulations. The climate change signal is computed as the difference between the historical experiment (1990–2014) and the SSP5-8.5 experiment (2076–2100), averaged across the ensemble. The realization code indicates the variant, initialization, physics, and forcing combination used for each model.

6 ERA5 Differences

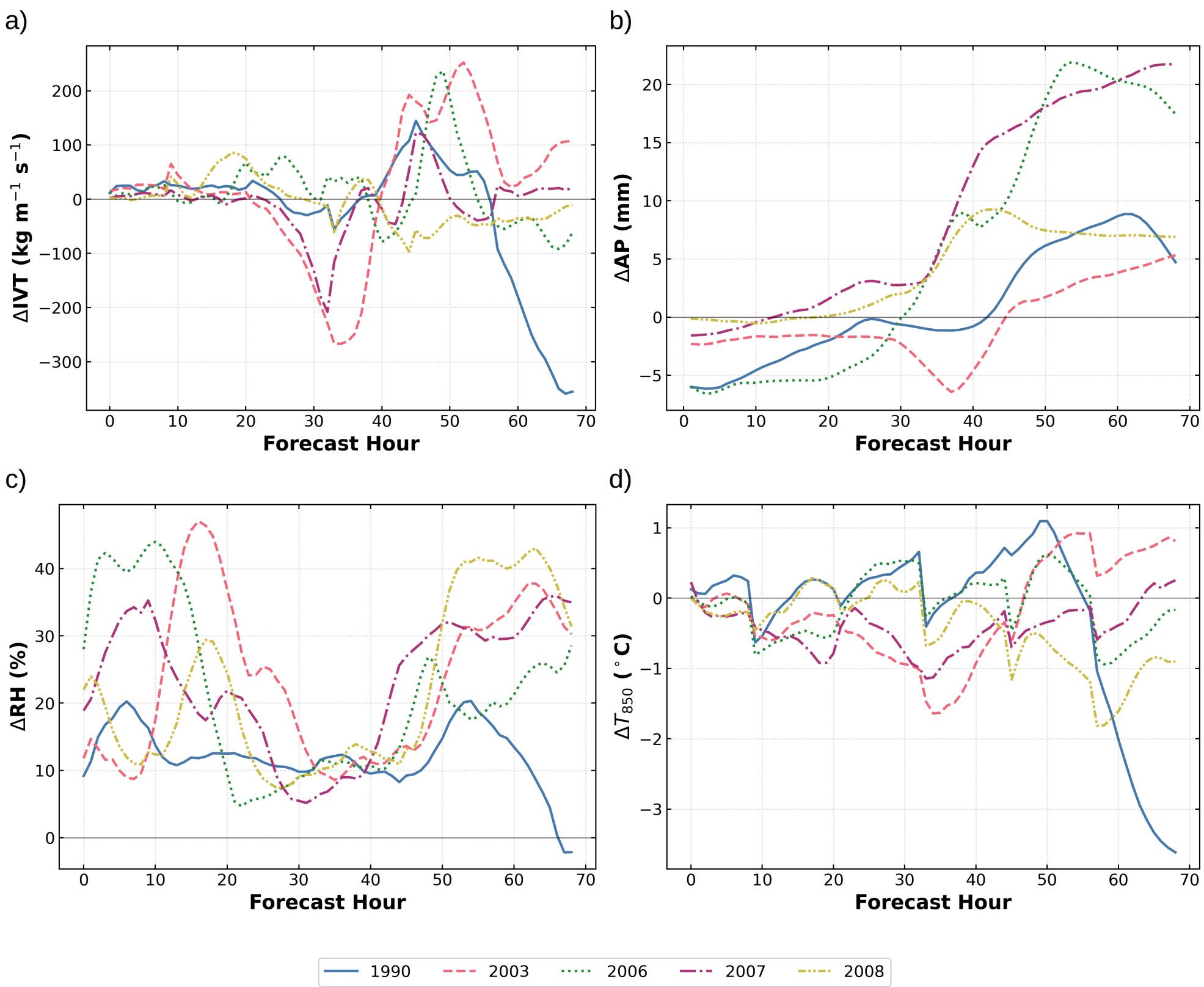

Fig. 8 Difference (CRCM6-GEM5 minus ERA5) in the regional-mean time series for the five selected AR events. Event years: 1990, solid blue; 2003, dashed red; 2006, dotted green; 2007, dash-dot purple; 2008, dash-dot-dot yellow. (a) ΔIVT ($kg\,m^{-1}\,s^{-1}$), (b) ΔAP (mm), (c) ΔRH (%), and (d) Δ850 hPa temperature (°C).
